# Osmotic Pressure Enables High-Yield Assembly of Giant Vesicles in Solutions of Physiological Ionic Strengths

**DOI:** 10.1021/acs.langmuir.3c00457

**Published:** 2023-04-06

**Authors:** Alexis Cooper, Vaishnavi Girish, Anand Bala Subramaniam

**Affiliations:** † Department of Chemistry and Biochemistry, University of California, Merced, Merced, California 95343, United States; ‡ Department of Bioengineering, University of California, Merced, Merced, California 95343, United States

## Abstract

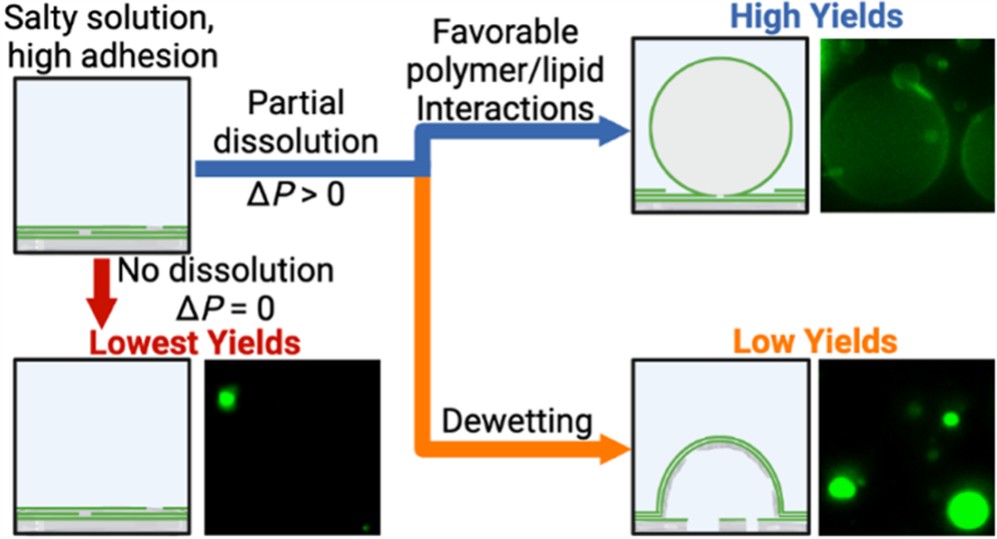

Giant unilamellar vesicles (GUVs) are micrometer-scale minimal cellular mimics that are useful for bottom-up synthetic biology and drug delivery. Unlike assembly in low-salt solutions, assembly of GUVs in solutions with ionic concentrations of 100–150 mM Na/KCl (salty solutions) is challenging. Chemical compounds deposited on the substrate or incorporated into the lipid mixture could assist in the assembly of GUVs. Here, we investigate quantitatively the effects of temperature and chemical identity of six polymeric compounds and one small molecule compound on the molar yields of GUVs composed of three different lipid mixtures using high-resolution confocal microscopy and large data set image analysis. All the polymers moderately increased the yields of GUVs either at 22 or 37 °C, whereas the small molecule compound was ineffective. Low-gelling temperature agarose is the singular compound that consistently produces yields of GUVs of greater than 10%. We propose a free energy model of budding to explain the effects of polymers in assisting the assembly of GUVs. The osmotic pressure exerted on the membranes by the dissolved polymer balances the increased adhesion between the membranes, thus reducing the free energy for bud formation. Data obtained by modulating the ionic strength and ion valency of the solution shows that the evolution of the yield of GUVs supports our model’s prediction. In addition, polymer-specific interactions with the substrate and the lipid mixture affects yields. The uncovered mechanistic insights provide a quantitative experimental and theoretical framework to guide future studies. Additionally, this work shows a facile means for obtaining GUVs in solutions of physiological ionic strengths.

## Introduction

Giant unilamellar vesicles, GUVs, vesicles with a single bimolecular wall of phospholipids with diameters ≥1 μm, mimic the minimal configuration of biological cells.^1,2^ GUVs are useful for applications in bottom-up synthetic biology and biomedicine.^3−12^ Assembling GUVs in solutions with ionic concentrations ∼100–150 mM Na/KCl (salty solutions) using thin-film hydration, however, is difficult.^1,13,14^ This limitation presents a longstanding challenge for the use of GUVs in applications since biomolecules such as proteins, nucleic acids, and polysaccharides require salty solutions to function.^15−19^

Several approaches have been proposed to improve the yields of GUVs in salty solutions. Soluble hexoses such as fructose,^20^ macromolecular polymeric films composed of ultralow-gelling temperature (ULGT) agarose,^21^ poly(vinyl alcohol) (PVA),^22^ cross-linked polyacrylamide,^23^ and cross-linked dextran (polyethylene glycol)^24^ have been used to assist the assembly of GUVs in salty solutions. The relative effectiveness of these various compounds compared to each other and the yields of GUVs that they produce, however, are unknown. Importantly, the lack of quantitative data hinders a physicochemical understanding of the mechanism through which these compounds exert their effects on the assembly of GUVs.

Here, we investigate the molar yields of GUVs in salty solutions as a function of the temperature and chemical identity of the assisting compounds. We performed experiments using three lipid mixtures, the zwitterionic phospholipid diolyeoyl-*sn*-glycero-3-phosphocholine (DOPC), a lipid mixture that minimally mimics the composition of the endoplasmic-reticulum-Golgi intermediate compartment (ERGIC) membrane,^25^ and a mixture that minimally mimics the phospholipid composition of the exoplasmic leaflet of the mammalian cellular membrane (mammalian exoplasmic leaflet (MEL)).^26,27^ DOPC and the MEL mixture are widely used in biophysical experiments.^28−31^ The ERGIC membrane is the location where transmembrane proteins including viral proteins such as SARS-CoV2 are inserted.^32−34^ We evaluated the small molecule fructose, the synthetic macromolecular polymer PVA, and four natural macromolecular polysaccharides, agaroses with varying gelling temperatures.

The yield of GUVs assembled in low-salt solutions without assisting compounds ranged from a low of 5% for the MEL mixture to a high of 25% for the ERGIC mixture. The yield of GUVs from the DOPC mixture was 17%. In salty solutions, the yields of GUVs for all of the lipid mixtures were very low, <1%. All of the polymeric compounds were able to assist the assembly of GUVs in salty solutions either at an incubation temperature of 22 or 37 °C. Apart from low-gelling temperature (LGT) agarose, all experiments with the polymers as assisting compounds resulted in only moderate to low yields of GUVs in salty solutions, ranging from 2 to 10%. Fructose was ineffective as an assisting compound, producing very low yields, <1%, at both temperatures.

We develop a free energy model of budding to explain the effects of macromolecular polymers on the yield of GUVs. High concentrations of ions increase the intermembrane adhesion potential between lipid bilayers in lamellar stacks. Our model shows that the gradient in osmotic pressure imposed by the dissolving polymer can balance the increased adhesion potential of the membranes in salty solutions. We test the model by experimentally modulating the adhesion potential using buffers of varying ionic strengths and ion valencies. Our results support the prediction that the osmotic pressure of the dissolving polymer acts to oppose adhesion between membranes. The polymers’ contribution to the osmotic pressure results in a net reduction in the free energy for bud formation.

Additionally, we find that interactions of the polymers with the substrate and with the lipid mixture affect yields. Dewetting of the polymer from the substrate results in the formation of polymer–lipid “pseudobuds” that morphologically resemble GUV buds but do not produce free-floating vesicles. When the moderately anionic low-gelling temperature agarose^35^ was used as the assisting compound, the yield of GUVs composed of the negatively charged ERGIC mixture decreased by 7 and 10%, respectively. Conversely, when the highly anionic PVA^36^ was used as the assisting compound, the yield of GUVs composed of the ERGIC mixture increased by 5% when compared to the DOPC and MEL mixtures. In aggregate, we find that low-gelling temperature agarose is the singular compound that consistently produces yields of GUVs of ≥10%. Partial dissolution of the polymer with minimal dewetting is essential for obtaining high yields of GUVs in salty solutions.

## Results and Discussion

### Properties of the Compounds Tested

**Figure 1:**
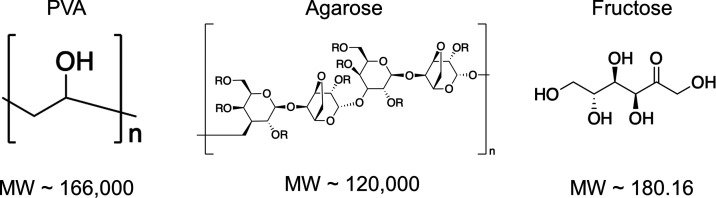
Structural formulas and molecular weights of the compounds. Structural formula of agarose^67^ R = H, CH_3_ or CH_2_CH_2_OH.^38^

Figure 1 shows the chemical structure and properties of the compounds that we tested. PVA is a water-soluble highly anionic synthetic polymer composed of vinyl monomer units.^36^ The PVA used in this study has a molecular weight of 146–186 kDa and is ≥99% hydrolyzed. Uncross-linked PVA at a concentration of 5 wt % or less does not gel and remains a viscous liquid at room temperature.^36^ Dehydrated PVA forms a partially soluble swollen polymer film when rehydrated below its glass transition temperature of ∼85 °C.^36^ The solubility of this polymer in water is 6% at 20 °C and 13% at 40 °C.^36^

Agarose is a naturally derived polysaccharide composed of 1,3-linked β-d-galactopyranose and 1,4-linked 3,6-anhydro-α-l-galactopyranose with an average molecular weight of 120 kDa.^37^ Solutions of agarose are conventionally prepared by dissolving 2 wt % or less of agarose powder at elevated temperatures.^38^ When the solution is cooled to below the “gelling” temperature, the agarose polymers transition from random coils into double helices.^38^ The double helices hydrogen bond to form a percolated gel network.^38^ Heating to above the “melting” temperature dissolves the gel into a liquid polymeric solution consisting of random agarose coils.^38^ Agarose exhibits thermal hysteresis. The melting temperature is significantly higher than the gelling temperature.^39^ Synthetic hydroxyethylation modifies the gelling and melting temperature of agarose.^35,38^ We refer to the agaroses by the manufacturer’s classification as ultralow gelling temperature (ULGT) agarose, low-gelling temperature (LGT) agarose, medium gelling temperature (MGT) agarose, and high gelling temperature (HGT) agarose. We show the gelling temperatures of the agaroses in Table S1, Supporting Information. We expect that ULGT, LGT, and MGT agarose to demonstrate partial solubility at room temperature, while HGT agarose is expected to be insoluble.

Fructose is a highly water-soluble small molecule monosaccharide with a molecular weight of 180.16 Da.^40^ Fructose is thus ∼600× smaller than PVA and agarose.

### Poorly Soluble HGT Agarose and Highly Soluble Fructose Are Ineffective at Assisting the Assembly of GUVs in Salty Solutions at 22 °C

We assembled GUVs composed of the zwitterionic lipid DOPC by hydrating the lipid-coated surfaces in a solution of phosphate buffered saline (PBS) + 100 mM sucrose at 22 °C (room temperature). This incubation temperature was above the gelling temperature of the ULGT agarose and below the gelling temperature of the LGT, MGT, and HGT agaroses. The sucrose was necessary to obtain a density gradient for sedimentation and is present in all the hydration buffers that we used. After 2 h of incubation, we harvested the vesicle buds from the surfaces and compared the molar yields of the resultant GUVs. The molar yield measures the moles of lipids in the membranes of the population of harvested GUVs relative to the moles of lipids that were initially deposited on the substrate.^41^ The molar yield is an objective measure that allows quantitative comparison of the effects of experimental variables on the yield of GUVs.^41^ Similar to our previous work, which used no assisting compounds, we allow the GUVs to sediment for 3 h prior to obtaining high-resolution single-plane open-pinhole tile scan images with a confocal microscope.^41^ We process the images in MATLAB and exclude non-GUV structures such as vesicles <1 μm in diameter, bright lipid aggregates, and multilamellar vesicles from the analysis.

We confirmed that the GUVs assembled with the use of assisting compounds showed similar sedimentation behavior to those assembled without any assisting compounds over the 3-h time frame (see Supporting Information Text and Figure S1). Thus, statistically significant differences in the measured molar yields are because of the effects of the assisting compounds and the experimentally controlled assembly conditions and not because of differences in sedimentation behavior or measurement technique. To account for experimental variation, experiments for each condition were repeated three independent times and the data are reported as the mean of the three independent repeats. We perform balanced one-way analysis of variance (ANOVA) tests to analyze the statistical significance of differences among multiple means. If the ANOVA revealed that at least one of the conditions had a significant effect on the molar yield, we performed Tukey’s Honestly significant difference (HSD) post hoc tests to determine the significance between pairs of conditions. We performed Student’s *t*-tests to compare two pairs of means. Tables of *F*- and *p*-values of the statistical tests are shown in Tables S2–S9 in the Supporting Information.

**Figure 2:**
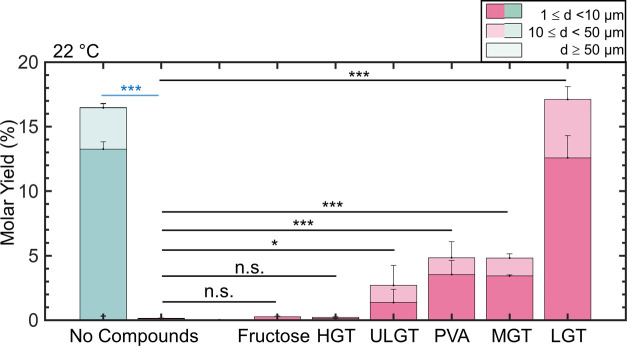
Stacked bar plots of the molar yields of GUVs at 22 °C. The blue bar is for samples hydrated in a low-salt solution consisting of 100 mM of sucrose. The pink bars are molar yields for samples hydrated in PBS with 100 mM sucrose. The two leftmost bars show the molar yield without any assisting compounds. Each bar is split into three regions corresponding to the diameter ranges specified in the legend. Statistical significance was determined using a balanced one-way ANOVA and Tukey’s HSD post hoc tests (black) and Student’s *t*-test (blue). *p < 0.05, **p < 0.01, ***p < 0.001, n.s. = not significant.

Figure 2 shows a stacked bar plot of the molar yields. We divide the yield data into small GUVs (diameters *d*, 1 μm ≤ *d* < 10 μm), large GUVs (10 μm ≤ *d* < 50 μm), and very large GUVs (*d* ≥ 50 μm). Error bars are the standard deviation from the mean. We show the histogram of the distribution of diameters in Figure S2 and representative images of the harvested vesicles in Figure S3.

To isolate the effects of the assisting compounds on assembly in salty solutions, we perform experiments on bare glass surfaces without any assisting compounds in PBS + 100 mM sucrose and in a low-salt buffer consisting only of 100 mM sucrose. This assembly condition has been referred to as gentle hydration or spontaneous swelling in the literature. At room temperature, the yield of GUVs from bare glass in a hydrating solution consisting of 100 mM of sucrose is 16.5 ± 0.3%. This result is consistent with previous reports for gentle hydration on glass surfaces.^41^ The yield of GUVs that we obtained from the lipids deposited on bare glass surfaces in the buffer containing PBS was ∼100 times lower, 0.2 ± 0.0% (*p* = 6.73 × 10^–8^), than the yield in buffer consisting only of sucrose.

We next assessed the effect of assisting compounds on the yields of GUVs in PBS. The use of fructose-doped lipid and HGT agarose as the assisting compounds resulted in a yield of 0.2 ± 0.0% and 0.3 ± 0.0%, which was statistically indistinguishable from bare glass (both *p* ≥ 0.999). The use of ULGT agarose, MGT agarose, and PVA as the assisting compounds resulted in statistically significant increases in the yields of GUVs compared to assembly without any assisting compounds (all *p* < 0.05). However, the yields were statistically indistinguishable from each other at 2.7 ± 0.2%, 4.8 ± 0.5%, and 5.0 ± 1.0%, respectively (all *p* > 0.05). The use of LGT agarose as the assisting compound resulted in the highest yield of GUVs at 17 ± 1%. This yield is more than three times higher than the yield of GUVs obtained when ULGT agarose and PVA are used as the assisting compounds. This result is notable since ULGT agarose and PVA are both used extensively in the literature,^42−50^ whereas, as far as we know, we are the first to report the use of LGT agarose. Our results are general. Use of ULGT and LGT agaroses with different catalog numbers as the assisting compounds resulted in similar yields to those shown in Figure 2 (Figures S4 and S5).

### Increase in the Incubation Temperature to 37 °C Increases the Yields of GUVs for Most of the Compounds But Decreases the Yield for LGT Agarose

**Figure 3:**
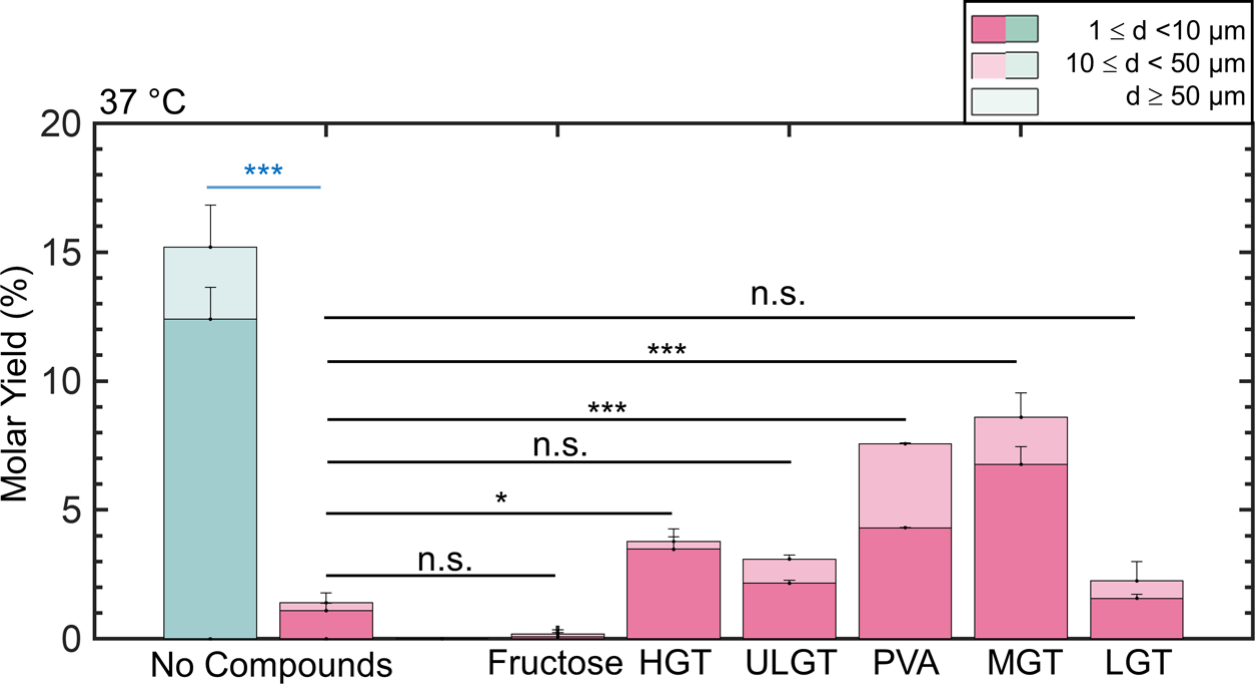
Stacked bar plots of the molar yields of GUVs at 37 °C. The blue bar is for samples hydrated in a low-salt solution consisting of 100 mM of sucrose. The pink bars are molar yields for samples hydrated in PBS with 100 mM sucrose. The two leftmost bars show the molar yield without any assisting compounds. Each bar is split into three regions corresponding to the diameter ranges specified in the legend. Statistical significance was determined using a balanced one-way ANOVA and Tukey’s HSD post hoc tests (black) and Student’s *t*-test (blue). *p < 0.05, **p < 0.01, ***p < 0.001, n.s. = not significant.

HGT agarose is the least soluble of the polymeric compounds tested, and fructose is the smallest and most soluble of the compounds tested. Both these compounds were ineffective at assisting the assembly of GUVs (Figure 2). Since the size and apparent solubility of the compounds appear to have an effect on the yields of GUVs, we devised an experiment to test for the effect of polymer solubility by assembling the GUVs at 37 °C. Naively, we expect that increasing the temperature should (1) enhance the yield of GUVs by increasing the solubility of the polymers and (2) have no effect on the yield of GUVs when the small molecule fructose is used as an assisting compound. Figure 3 shows the results of our experiments. We show representative images of the harvested GUVs in Figure S6 and the histogram of the distribution of diameters in Figure S7.

An increase in temperature could in principle increase the yields of GUVs independent of any effects of the assisting compounds. We thus performed experiments to determine the effect of the increase in temperature on the yield of GUVs without any assisting compounds. The yield of GUVs on bare glass in the low-salt sucrose buffer was 15 ± 1%. This yield was statistically indistinguishable from the assembly at room temperature. Conversely, the increased temperature resulted in a modest but statistically significant increase in the yields of GUVs on bare glass in PBS to 1.4 ± 0.4% (*p* = 0.00480).

The use of PVA, MGT agarose, and HGT agarose as the assisting compounds resulted in statistically significant increases in the yield of GUVs compared to assembly performed at room temperature, 7.6 ± 0.8% (*p* = 0.0314), 9.0 ± 0.3% (*p* = 0.0224), and 3.8 ± 0.5% (*p* = 2.20 × 10^–4^), respectively. The yield of GUVs from the fructose-doped lipid was unaffected by the change in temperature. These four results are consistent with our naïve expectations of the effect of temperature on the yields of GUVs.

The use of ULGT agarose as the assisting compound resulted in no change in yields at 3.1 ± 0.1%, while the fuse of LGT agarose as the assisting compound showed an almost 9-fold decrease in the yield of GUVs to 2.0 ± 0.1% (*p* = 1.40 × 10^–5^). This dramatic drop in yield for LGT agarose resulted in the yields of GUVs from the samples prepared with ULGT agarose and LGT agarose as the assisting compounds to be statistically indistinguishable from simple gentle hydration in PBS at 37 °C. Note that 37 °C was above the gelling temperature of ULGT and LGT agarose. We conclude that assembly at temperatures exceeding the gelling temperature of the agarose results in dramatically lowered yields of GUVs.

The increase in the yield of GUVs with the increase in temperature when PVA, MGT agarose, and HGT agarose are used as assisting compounds shows the importance of the solubility of the polymer for assisting in the assembly of GUVs in salty solutions. The decrease in the yield of GUVs when LGT agarose is used as the assisting compound, and the unchanged yields when ULGT agarose is used as the assisting compound, however, show that additional factors play a role.

### Characterization of the Hydrated Lipid Films Reveals the Formation of Polymer–Lipid Pseudobuds due to the Dewetting of the Polymer

**Figure 4:**
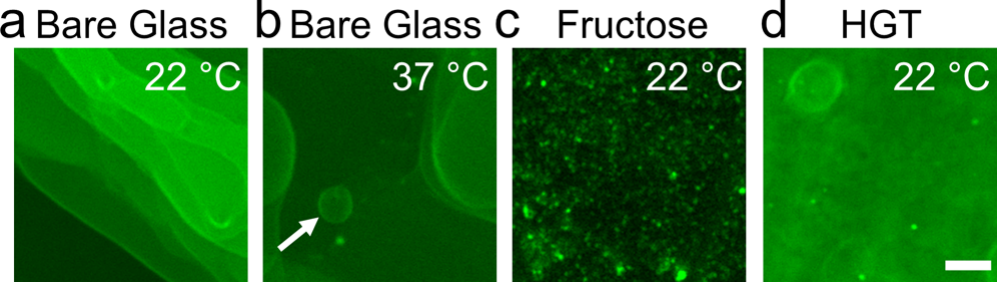
High-resolution confocal images of surfaces that have little to no spherical buds. All of the samples were hydrated in PBS with 100 mM sucrose. (a) Bare glass at 22 °C, (b) bare glass at 37 °C. The white arrow points to an example of a spherical bud. (c) Fructose at 22 °C, (d) HGT agarose at 22 °C. The scale bar is 15 μm.

To explore potential factors that could explain our results, we imaged the lipid-coated surfaces prior to harvesting using high-resolution single-plane confocal microscopy. On bare glass coverslips, we observe flat fluorescent surfaces with stepped differences in intensity and few spherical buds (Figure 4a). These images are reminiscent of supported lipid bilayers on glass surfaces.^51^ We interpret the stepped difference in fluorescence intensity as overlapping bilayers in a stack. Compared to surfaces incubated at room temperature, spherical buds can be seen in typical fields of views on the surfaces incubated at 37 °C (Figure 4b, white arrow). Images of the fructose-doped lipid did not show any regions that appeared to be lipid bilayers or vesicle buds (Figure 4c). Instead, the surface was characterized by irregular punctate structures. We interpret these structures as being lipid aggregates. The lipid film appeared smooth on HGT agarose at room temperature (Figure 4d).

**Figure 5:**
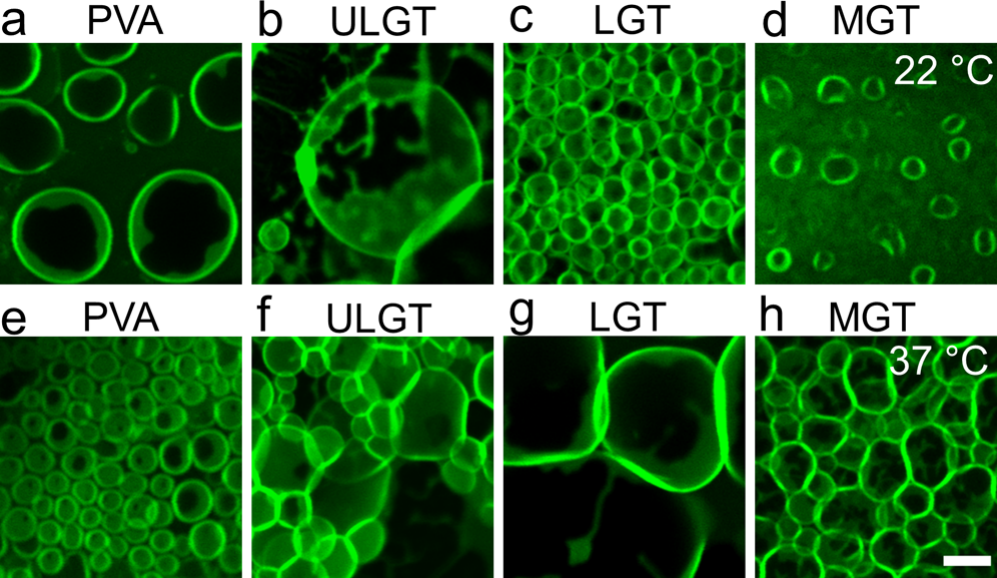
High-resolution confocal images of surfaces that have spherical buds. The top row shows images from samples hydrated at 22 °C. The bottom row shows images from samples hydrated at 37 °C. All samples hydrated in PBS with 100 mM of sucrose. The scale bar is 15 μm.

We find a high density of spherical structures reminiscent of GUV buds on all the other polymer-coated surfaces (Figure 5). Similar to assembly without assisting compounds,^41^ the GUV buds remain attached to the surface prior to harvesting (Figure S8). Puzzlingly, surfaces that appeared to have a high density of large spherical buds such as PVA-coated surfaces and ULGT agarose-coated surfaces at room temperature and ULGT, LGT, and MGT agarose-coated surfaces at 37 °C had relatively low yields of free-floating GUVs. Furthermore, these surfaces often had buds of larger diameters and of higher surface densities compared to the high yielding LGT agarose-coated surfaces at room temperature.

**Figure 6:**
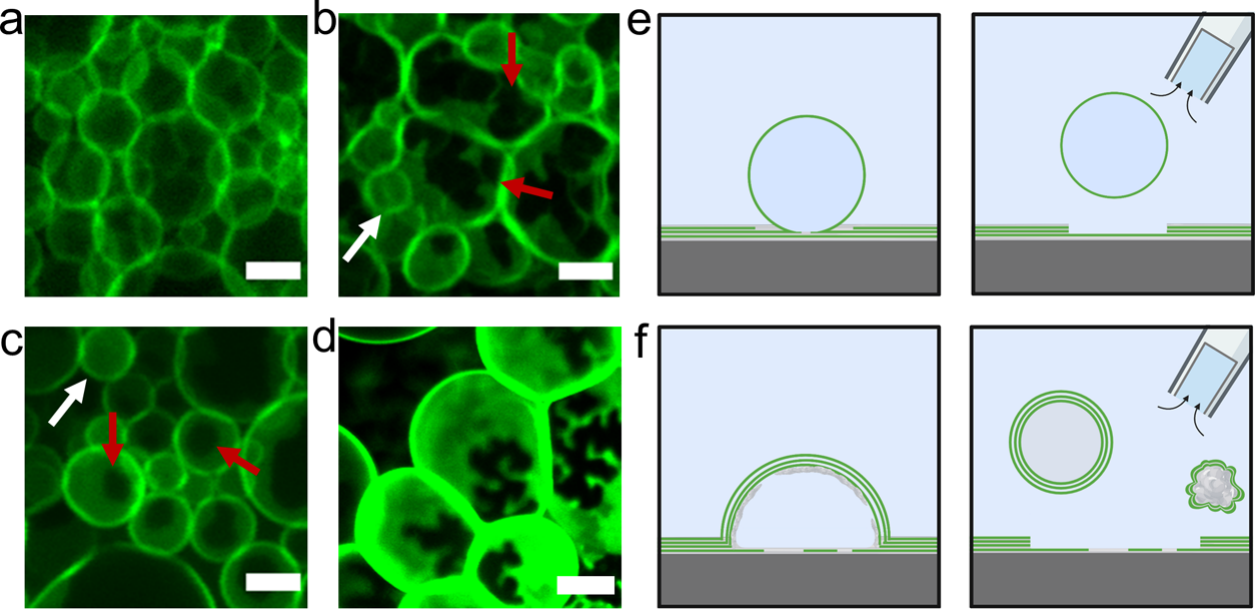
Polymer–lipid pseudobuds as well as GUV buds form on polymer-coated surfaces in salty solutions. (a–d) High-resolution confocal images of the surfaces. (a) Spherical GUV buds with uniform fluorescence intensity on LGT agarose hydrated in 100 mM sucrose. (b) Polymer–lipid pseudobuds with spinodal dewetting patterns on LGT agarose are highlighted by the red arrows. For comparison, a GUV bud is highlighted by the white arrow. (c) Polymer–lipid pseudobuds with circular dewetting patterns on PVA (red arrows). For comparison, a GUV bud is highlighted by the white arrow. (d) Large polymer–lipid pseudobuds with high fluorescence intensity on ULGT agarose. (e–d) Schematic showing the different outcomes for harvesting GUV buds or polymer–lipid pseudobuds from the surfaces. (e) GUV buds close to form isolated GUVs when harvested from the surface. (f) Polymer–lipid pseudobuds form non-GUV structures such as lipid-coated polymer aggregates. (b–d) Hydrated in PBS with 100 mM sucrose. The scale bars for (a)–(c) are 10 μm. The scale bar for (d) is 20 μm.

To understand further why surfaces with high numbers of apparent buds showed low yields of free-floating GUVs, we evaluated the structure of the buds by examining the distribution of fluorescence intensity. GUV buds imaged using confocal microscopy have a uniform fluorescence intensity.^41^ Consistent with this previous observation, all the buds on surfaces that were incubated in low-salt solutions have a uniform distribution of fluorescence intensities (Figure 6a). On the polymer-coated surfaces in PBS, we find two types of structures. Buds with uniform fluorescence intensities reminiscent of GUV buds (white arrows in Figure 6b,c) and novel buds with dark regions of low fluorescence intensity (red arrows in Figure 6b,c). These dark regions had shapes that could be spinodal-like (red arrows in Figure 6b) or circular (red arrows in Figure 6c). Further, buds that showed these patterns often were larger and had higher membrane fluorescence intensities compared to the buds without dark regions (compare Figure 6a with Figure 6d).

Based on these observations, we propose that on polymeric surfaces, two types of buds form (Figure 6e,f). The first are regular GUV buds that self-close to form free-floating GUVs when scissioned from the surface during harvesting (Figure 6e).^41^ The second are hybrid polymer–lipid “pseudobuds” that nominally resemble GUV buds. We propose that the polymer–lipid pseudobuds arise when the lipid-coated polymer film swells and dewets from the surface (Figure 6f). This interpretation explains the dark region at the base of the pseudobuds where the polymer has lifted with the lipid. Indeed, the spinodal and circular patterns at the base of the pseudobuds are reminiscent of spinodal and heterogeneous nucleation patterns observed for polymer and polysaccharide films that dewet from a supporting substrate.^52−55^ Localized dewetting of the polymer film and lifting of the stacks of lipids also explain the high membrane fluorescence intensity of the pseudobuds. The high fluorescence intensity suggests that pseudobuds are composed of multiple lipid bilayers. We posit that the layer of dewetted polymer that scaffolds the pseudobuds prevents closure of the multibilayer lipid membrane to form vesicles when scissioned from the surface during harvesting (Figure 6f). Furthermore, even if the buds self-close, the multiple bilayers and the high amount of encapsulated polymer make these objects not GUVs.

**Figure 7:**
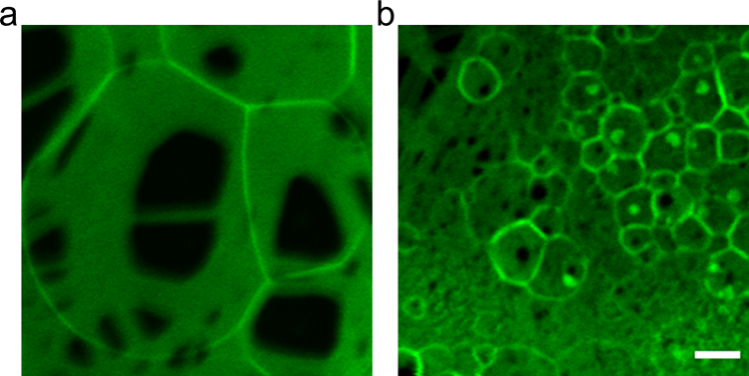
DOPE forms pseudobuds on ULGT agarose and PVA. (a) Pseudobuds formed from DOPE on ULGT agarose showing regions of dewetting with low fluorescence intensity. (b) Pseudobuds formed from DOPE on PVA showing dewetting patterns. DOPE does not form lamellar structures, so these buds are not GUV buds. Samples were hydrated in PBS with 100 mM of sucrose. The scale bar is 10 μm.

We test for the formation of polymer–lipid pseudobuds using the lipid 1,2-dioleoyl-*sn*-glycero-3-phosphoethanolamine (DOPE). DOPE cannot assemble into vesicles because it forms hexagonal phases instead of lamellar phases.^56^ We thus expect no GUV buds to form on the surface. We use PVA and ULGT agarose, two polymers that likely cause the formation of high numbers of pseudobuds. Our images show clear structures resembling pseudobuds, and no structures resembling GUV buds (Figure 7). On harvesting, we obtained no measurable yield of GUVs. We conclude that films of lipids and polymers can form pseudobuds that nominally resemble GUV buds. These pseudobuds are not productive for forming GUVs.

Analysis of our images showed that the number of pseudobuds was low for the LGT agarose-coated surface at room temperature, explaining the high yield of free-floating GUVs. At 37 °C, which is above the gelling temperature of LGT agarose, the number of pseudobuds increases at the expense of GUV buds. The increased formation of pseudobuds due to dewetting explains the low yield of free-floating GUVs. All of the other polymer surfaces have a high number of pseudobuds. Thus, although the surfaces of polymers can appear to be covered with a large number of spherical buds (Figure 5), most of the buds cannot be harvested to form GUVs. Our discovery of pseudobuds explains previous observations of low yields of free-floating GUVs despite the apparent high numbers of spherical buds on the surfaces.^13,22^ We surmise that dewetting of the polymer acts antagonistically to polymer solubility and reduces the yields of GUVs.

### Membrane Composition Modifies the Yields of GUVs Obtained from LGT Agarose and PVA

**Figure 8:**
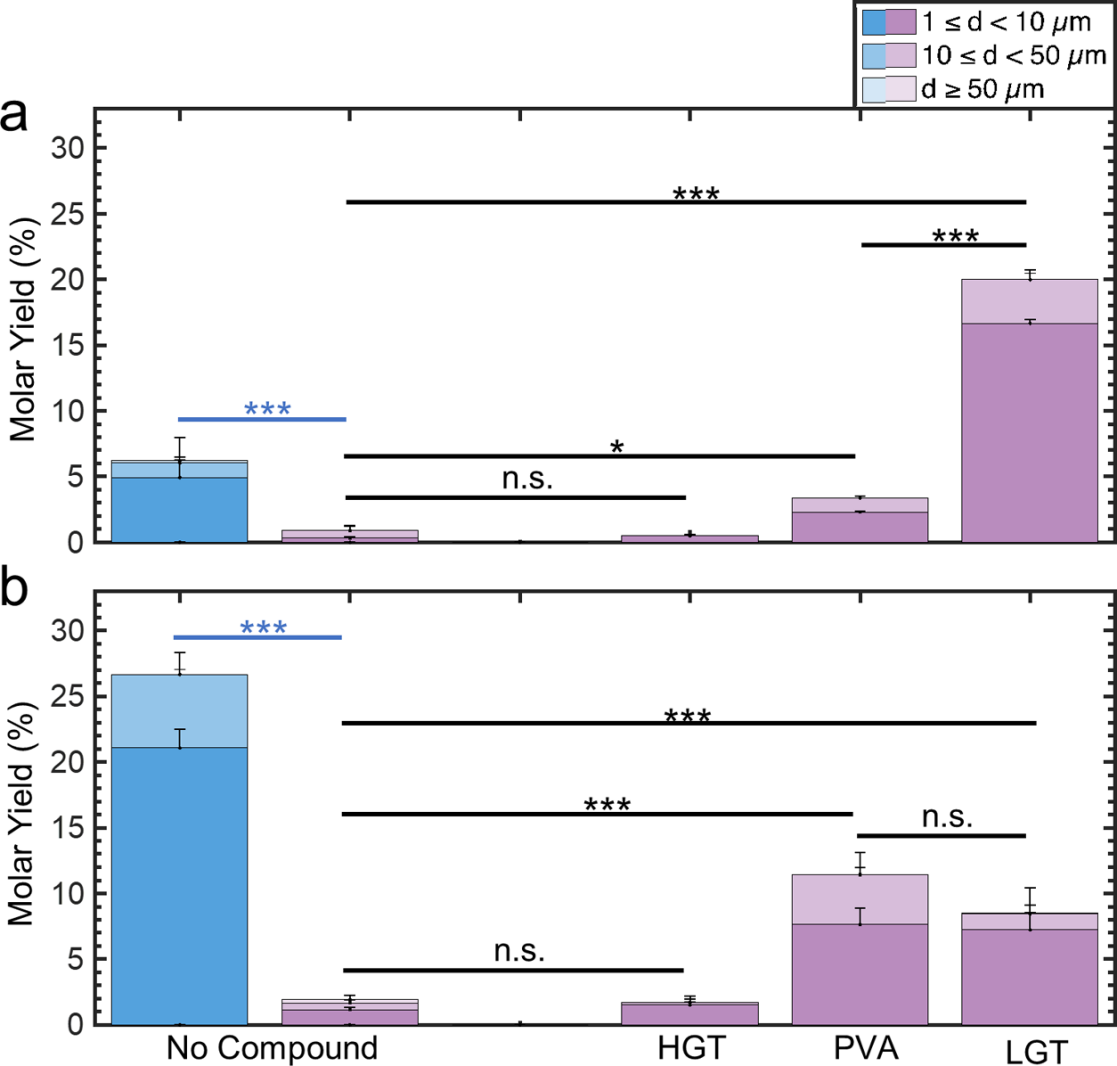
Lipid composition has an effect on the molar yields of GUVs. The blue bar is for samples hydrated in a low-salt solution consisting of 100 mM of sucrose. The purple bars are molar yields for samples hydrated in PBS with 100 mM sucrose. (a) MEL mixture. The two leftmost bars show the molar yield without any assisting compounds. (b) ERGIC mixture. The two leftmost bars show the molar yield without any assisting compounds. Each bar is split into three regions corresponding to the diameter ranges specified in the legend. Statistical significance was determined using a balanced one-way ANOVA and Tukey’s HSD post hoc tests (black) and Student’s *t*-test (blue). *p < 0.05, **p < 0.01, ***p < 0.001, n.s. = not significant.

We studied the effect of three assisting compounds, PVA, LGT agarose, and HGT agarose on the yields of GUVs obtained using an ERGIC mimicking mixture and a MEL mimicking mixture. The ERGIC mixture is negatively charged since it contains a high mol fraction of phosphatidylserine and phosphatidylinositol. Figure 8 shows the results of our experiments. We show the histogram of the distribution of diameters in Figures S9 and S10.

Similar to our approach with DOPC, we first measured the yield of GUVs obtained in low-salt and salty solutions without any assisting compounds. In low-salt solutions, we obtained a significantly lower yield of GUVs composed of the MEL mixture compared to the DOPC mixture, 6.2 ± 1.8% (*p* = 6.07 × 10^–4^), and a significantly higher yield of GUVs composed of the ERGIC mixture compared to the DOPC mixture, 27 ± 1.7% (*p* = 4.98 × 10^–4^). These results show that in low-salt solutions, the yield of GUVs depends on the composition of the membrane. Both the MEL mixture and ERGIC mixture had very low yields of GUVs, 0.8 ± 0.3% and 1.9 ± 0.4%, respectively, in salty solutions. We conclude that assembly in salty solutions without any assisting compounds results in universally low yields of GUVs, < 2%.

In salty solutions, the use of LGT agarose as the assisting compound resulted in an increase in the yield of GUVs from the MEL mixture to 20 ± 0.8%, which was significantly higher than the yield obtained without the use of assisting compounds (*p* = 1.46 × 10^–13^). The use of PVA as the assisting compound resulted in a more modest yet statistically significant increase in the molar yield of GUVs composed of the MEL mixture to 3.4 ± 0.1% (*p* = 4.28 × 10^–4^). Similar to our results with DOPC, the use of HGT agarose as the assisting compound did not result in an increase in the yield of GUVs composed of the MEL mixture compared to bare glass, 0.9 ± 0.3% (*p* = 0.738).

The use of LGT agarose and PVA as the assisting compounds resulted in statistically significant increases in the yield of GUVs composed of the ERGIC mixture to 8.5 ± 1.9% (*p* = 1.30 × 10^–3^) and 11 ± 1.7% (*p* = 9.83 × 10^–5^), respectively. The difference in the mean yields of GUVs obtained between the two polymers was not statistically significant. Interestingly, the effect of the membrane composition and the chemical identity of the polymer was significant. When LGT agarose was used as the assisting compound, the resulting yields of GUVs composed of the ERGIC mixture were lower by 9% (*p* = 5.46 × 10^–4^) and 12% (*p* = 1.08 × 10^–4^) when compared to the zwitterionic DOPC and MEL mixtures, respectively. Conversely, when PVA was used as the assisting compound, the resulting yields of GUVs composed of the ERGIC mixture increased by 6% (*p* = 1.33 × 10^–3^) and 8% (*p* = 4.43 × 10^–4^) when compared to the zwitterionic DOPC and MEL mixtures, respectively. Similar to the DOPC and MEL mixtures, the use of HGT agarose as the assisting compound had no significant effect on the yield of GUVs composed of the ERGIC mixture, 1.7 ± 0.5% (*p* = 0.998).

We surmise that in salty solutions, the poorly soluble HGT agarose was ineffective at increasing yields for all lipid mixtures, while the soluble LGT agarose and PVA increased yields for all lipid mixtures. Additionally, the membrane composition, likely the anionic nature of the ERGIC membrane, causes polymer-specific changes in the yields of GUVs.

### Osmotic Pressure Exerted by Dissolving Polymers Assists the Assembly of GUVs

Currently, there is no consensus on how assisting compounds promote the assembly of GUVs.^13,20,22,57^ Upon hydration, lipids assemble into multibilayer stacks that conform to the geometry of the supporting solid substrate.^58^ We had previously shown that in the absence of assisting compounds, gentle hydration of lipid films on surfaces composed of nanoscale cylindrical fibers (using nanocellulose paper in the Paper-Abbetted amPhiphile hYdRation in aqUeous Solutions (PAPYRUS) method) resulted in twice the yields of GUVs compared to flat surfaces.^41^ We explained this result by showing that the free energy change for the formation of spherical buds from membranes templated on cylindrical fibers was lower than the free energy change from membranes templated on flat surfaces. In conditions where the energy to perform work is fixed, processes with low positive changes in free energy or high negative changes in free energy result in high yields of GUVs.^41^

Our results here show that on flat substrates, polymers that have partial solubility such as ULGT agarose, LGT agarose, MGT agarose, and PVA at 22 °C and HGT agarose at 37 °C can increase the yields of GUVs in salty solutions relative to bare glass. Dewetting of the soluble polymers from the surface, on the other hand, favors the formation of pseudobuds and reduces the yields of GUVs. Further, when compared to the zwitterionic DOPC and MEL mixtures, the yield of GUVs from the anionic ERGIC mixture is enhanced when the highly anionic PVA is used as an assisting compound and is decreased when LGT agarose is used as an assisting compound. Clearly, interactions between the assisting polymers with the solid glass support and the membrane can disfavor or enhance the formation of GUV buds. Consistently, we find that in conditions where the polymer has low solubility such as when HGT agarose is used as the assisting compound at room temperature, the yields of GUVs remain unchanged compared to bare glass (Figures 2 and 8). To understand the mechanistic importance of polymer dissolution on the formation of buds, we examine eq 1.

Equation 1 shows the change in free energy for forming a spherical bud from an initially flat bilayer, Δ*E*, retaining the pressure-volume term and dropping the edge energy term (see Supporting Information Text for further details).
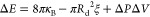
1

In this equation, κ_B_ is the bending rigidity of the membrane, *R*_d_ is the radius of the flat lipid disk that forms the spherical GUV bud, ξ is the adhesion potential between the membranes in a stack, Δ*P* is the difference in osmotic pressure, and Δ*V* is the difference between the volume of the spherical bud and the interlamellar volume enclosed by a putative disk of the equivalent area to the bud in the stack. ξ is negative for attractive interactions. In the absence of an osmotic pressure, that is Δ*P* = 0, the free energy change for the formation of buds is always positive.^41^ Energy due to hydrodynamic flows or temperature gradients^59^ provides work to form GUV buds.

Our data show that the yield of GUVs in low-salt solutions depends on the composition of the lipid membrane. This result is consistent with the expected differences in membrane properties such as adhesion and bending rigidity due to differences in composition (Figures 2 and 8). Our data also show that in salty solutions, the yields of GUVs from all three lipid compositions that we tested is very low (Figures 2, 3, and 8). This result suggests that the energy due to flows and temperature gradients, which was sufficient to produce high yields of GUVs in low-salt solutions, is insufficient to assemble GUVs in salty solutions. Dissolved ions increase adhesion between surfaces in aqueous solutions by screening electrostatic charges.^56,60^ Using characteristic values of adhesion energy of ξ = 1 × 10^–6^ J m^–2^ for DOPC membranes in low-salt solutions, ξ = 1 × 10^–4^ J m^–2^ in salty solutions, κ_B_ = 8.5 × 10^–20^ J, and *R*_d_ = 1.0 μm, we obtain that in low-salt solutions Δ*E* = 1284 *k*_B_*T* and in salty solutions Δ*E* = 76 958 *k*_B_*T*. The increase in the magnitude of the energy to form buds due to electrostatic screening is expected to decrease the number of buds formed. This result is consistent with our observed dramatic decrease in the yield of GUVs obtained through gentle hydration without assisting compounds in PBS compared to low-salt solutions (the leftmost bars in Figures 2, 3, and 8).

For polymer-coated surfaces, the dissolution of the polymer can create a difference in osmotic pressure in the interlamellar space of the bilayer stacks. The osmotic pressure acts in the opposite direction to the adhesion potential since there is a high concentration of polymers on the surface and no polymers in the bulk solution. Thus, Δ*P* is non-zero and negative. The negative third term on the right-hand side of eq 1 allows the possibility for no change or even a net decrease in free energy that can balance an increase in the magnitude of the adhesion potential. Our estimates show that the concentration of polymer in the interlamellar space with a distance of 4 nm in a stack consisting of five bilayers is approximately 2.6 M. Approximately 0.009% of the polymer molecules must dissolve in the interlamellar space, 0.25 mM, for the contribution of the osmotic pressure to result in similar budding energies between the polymer-free low-salt solutions and the polymer-assisted salty solutions (see Supporting Information Text for further discussion).

Since it is challenging to measure the dissolution of small amounts of polymer, we devise experiments to probe for the effects of osmotic pressure by modulating the magnitude of the adhesion potential of the membranes relative to PBS. The adhesion potential between membranes is lowest in solutions of low ionic strength. High concentrations of monovalent ions reduce the double layer screening length,^56,60^ while mM concentrations of divalent cations can neutralize surface charges or function as ionic bridges.^61,62^ These conditions promote adhesion between membranes^60,63,64^ (see Supporting Information Text, Tables S10 and S11 for further discussion and calculations of the double layer screening length). We reduced the adhesion potential relative to PBS by using 100 mM sucrose with no added salts and increased the adhesion potential by using 600 mM NaCl, a solution with a monovalent salt concentration that is more than 4 times that of PBS. We also used buffers with millimolar amounts of the divalent cations Mg^2+^ and Ca^2+^, PBS + 5 mM MgCl_2_ and 150 mM KCl + 5 mM CaCl_2_. The combinations of salts served to test for generality.

**Figure 9:**
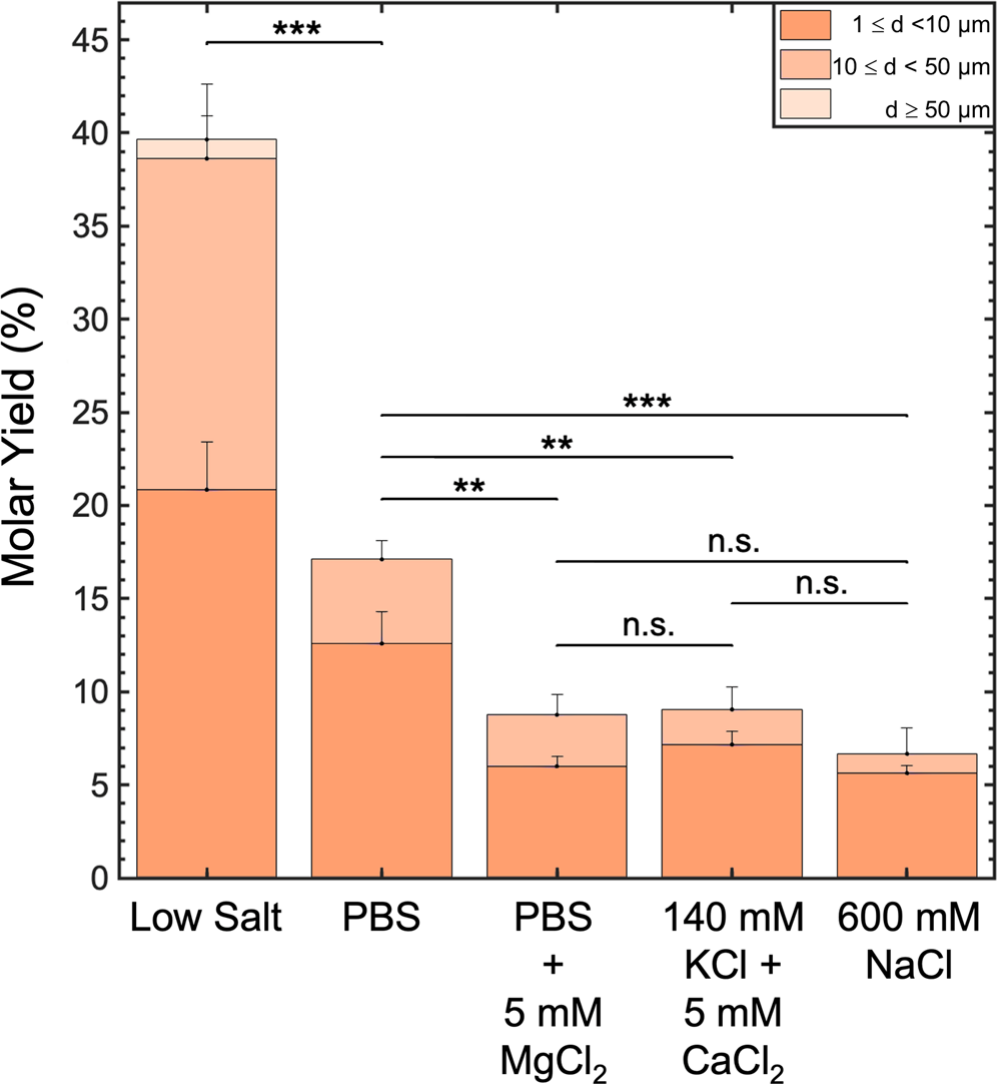
Yield of GUVs depends on the concentration and valency of ions. Stacked bar plot showing yields of GUVs assembled on LGT agarose hydrated in solutions containing low salt, PBS, PBS + 5 mM MgCl_2_, 140 mM KCl + 5 mM CaCl_2_, and 600 mM NaCl. The PBS data are reproduced from Figure 2. Each bar is split into three regions corresponding to the diameter ranges specified in the legend. Each bar is the average of three samples. Statistical significance was determined using a one-way ANOVA and Tukey’s HSD post hoc tests. *p < 0.05, **p < 0.01, ***p < 0.001, n.s. = not significant. All the buffers contained 100 mM of sucrose.

Figure 9 shows the molar yields of GUVs obtained when LGT agarose was used as the assisting compound. We show the histogram of the distribution of diameters in Figure S11. The yield of GUVs was affected significantly by the composition of the hydrating buffer. In solutions devoid of salt, the yield of GUVs doubled to 40 ± 3% compared to assembly in PBS (*p* = 1.43 × 10^–7^). In 600 mM NaCl, PBS + 5 mM MgCl_2_, and 150 mM KCl + 5 mM CaCl_2_, the yield was approximately halved to 6.0 ± 1.0% (*p* = 8.14 × 10^–5^), 9.0 ± 1.0% (*p* = 0.00124), and 9.0 ± 1.0% (*p* = 0.00137), respectively. Clearly, buffers that decrease the adhesion potential result in an increase in the yield, while buffers that increase the adhesion potential result in a decrease in the yield. These results are consistent with the prediction that the osmotic pressure of the dissolving polymers assists in the assembly of GUVs on polymer-coated surfaces. Since the dissolution of the polymer appears to be key for the formation of GUVs in salty solutions, extrapolating from our results for HGT agarose, cross-linking polymers to minimize dissolution will likely result in low yields of GUVs in salty solutions.

Our model shows that the dissolution of the polymer is sufficient in principle to cause the formation of GUV-sized buds in salty solutions. The concentration of polymer in the interlamellar volume is an important parameter. In all samples, the lipid that is dissolved in an organic solvent is deposited onto the dry polymer films. The rearrangement of the polymer and lipid in the transient milieu of the evaporating organic solvent and the subsequent hydration of the dry polymer/lipid film in the aqueous solvent to form lipid stacks interspersed with polymers likely determines the efficiency of the osmotic pressure mechanism. It is reasonable that the chemical composition of the polymer such as the presence of hydrophobic groups or charged groups affects the amount of polymer that incorporates in the interlamellar space of the lipid stack. Small molecule sugars are ineffective at increasing yields compared to simple gentle hydration on bare glass. The lack of effect of small sugars on yields that we find here is consistent with data from previous reports^20^ (Supporting Information Text). We suggest that the high solubility of sugars and their small size allow them to escape through defects in the bilayer stacks making sugars unable to exert an osmotic pressure against the membrane.

## Conclusions

Quantitative experiments reveal that the formation of GUVs from films of lipids in salty solutions depends significantly on the chemistry of the compounds, the assembly temperature, and the composition of the lipid membrane. The use of LGT agarose at room temperature as an assisting compound consistently resulted in the highest yields of free-floating GUVs in salty solutions for all the lipid mixtures tested. The use of other polymers as assisting compounds resulted in moderate to low yields of GUVs. Experiments with solutions of varying ionic strengths show that the difference in osmotic pressure due to dissolving polymers promotes the assembly of GUVs. Although the partial dissolution of the polymer is essential for increasing yields, specific interactions of the polymer with the substrate and the lipids can influence the yield. These results demonstrate the importance of measuring quantitative yields when novel assisting compounds, lipid mixtures, or temperatures are used to assist the assembly of GUVs. Looking forward, we propose that our quantitative experimental framework and our minimal free energy model provide a mechanistic guide for rational studies for discovering novel polymers that can further improve yields of giant vesicles in salty solutions, for example, from amphiphilic block copolymers.^65,66^

## Methods

### Materials

We purchased glass coverslips (Corning, 22 mm × 22 mm) and premium plain glass microscope slides (75 mm × 25 mm) from Thermo Fisher Scientific (Waltham, MA).

### Chemicals

We purchased sucrose (BioXtra grade, purity ≥99.5%), glucose (BioXtra grade, purity ≥99.5%), potassium chloride (molecular biology grade, ≥99.0%), magnesium chloride (purity ≥98%), casein from bovine milk (BioReagent grade), agarose type IX-A: ultralow-gelling temperature (catalog number: A2576, molecular biology grade), agarose: low-gelling poInt (catalog number: A9414, molecular biology grade), agarose type II-A medium EEO (catalog number: A9918), agarose type VI-A: high gelling temperature (catalog number: A7174), ultralow-gelling temperature agarose (catalog number: A5030), low-gelling temperature agarose (catalog number: A0701) and poly(vinyl alcohol) (*M*_W_ 146,000–186,000 99+% hydrolyzed) (PVA) from Sigma-Aldrich (St. Louis, MO). We purchased chloroform (ACS grade, purity ≥99.8%, with 0.75% ethanol as preservative), Invitrogen 10× PBS buffer (pH 7.4, 0.2 μm filtered, 1.37 M sodium chloride, 0.027 M potassium chloride, 0.080 sodium phosphate dibasic, 0.020 M potassium phosphate monobasic), sodium chloride (BioXtra grade, purity ≥99.5%) and d-(−)-fructose (high-performance liquid chromatography (HPLC) grade, purity ≥99%) from Thermo Fisher Scientific (Waltham, MA). We obtained 18.2 MΩ ultrapure water from an ELGA Pure-lab Ultra water purification system (Woodridge, IL). We purchased 1,2-dioleoyl-*sn*-glycero-3-phosphocholine (18:1 (Δ9-*cis*) PC (DOPC)), 23-(dipyrrometheneboron difluoride)-24-norcholesterol (TopFluor-Chol), 1,2-dioleoyl-*sn*-glycero-3-phosphoethanolamine (DOPE), 1-palmitoyl-2-oleoyl-glycero-3-phosphocholine (POPC), cholesterol (ovine wool, >98%), l-α-phosphatidylinositol (Liver, Bovine) (sodium salt) (Liver-PI), 1-palmitoyl-2-oleoyl-*sn*-glycero-3-phospho-l-serine (sodium salt) (POPS), and 1,2-distearoyl-*sn*-glycero-3-phosphoethanolamine-*N*-[methoxy(polyethylene glycol)-2000](ammonium salt) (PEG2000-DSPE) from Avanti Polar Lipids, Inc. (Alabaster, AL).

### Lipid Mixtures

Lipid mixtures were prepared as previously described with minor adaptations.^41^ Briefly, we prepared working solutions of DOPC/PEG2000-DSPE/TopFluor-Chol at 96.5:3:0.5 mol %, DOPE/PEG2000-DSPE/TopFluor-Chol at 96.5:3:0.5 mol %, POPC/Chol/PEG2000-DSPE/TopFluor-Chol at 66.5:29:3:0.5 mol %, and POPC/POPE/Liver-PI/POPS/Chol/PEG2000-DSPE/TopFluor-Chol at 41.5:20:13:7:15:3:0.5 mol % at a concentration of 1 mg mL^–1^. All lipid solutions were stored in Teflon-capped glass vials, purged with argon, and stored in a −20 °C freezer. Lipid solutions were remade weekly.

### Formation of Polymer Films on Glass

Films of polymer on glass coverslips (Corning, 22 mm × 22 mm) were prepared by applying 300 μL of 1 wt % (w/w) polymer on a coverslip and evenly spreading the solution with the side of a pipette tip.^66^ The coated glass coverslips were allowed to dehydrate on a hotplate for a minimum of 2 h set at a temperature of 40 °C. At the end of the process, the coverslip appeared flat and clear.

### Deposition of Lipids

To ensure standardized conditions that allow comparison between samples, we deposit lipid solutions as described previously.^41^ Briefly, circular disks with a diameter of 9.5 mm were traced on the underside of the polymer-coated coverslip or bare coverslips using a template made from a circle hole punch (EK Tools Circle Punch, 3/8 in.). We evenly deposited 10 μL of the lipid working solution onto the polymer-coated or bare side of the glass within the traced area using a glass syringe (Hamilton). All lipid-coated substrates were placed into a laboratory vacuum desiccator for 1 h to remove any traces of organic solvent before hydration.

### Procedure for Assembly

Circular poly(dimethylsiloxane) (PDMS) gaskets (inner diameter × height = 12 × 1 mm^2^) were affixed to bare coverslips or polymer-coated coverslips to construct a barrier around the dry solvent-free lipid films. We added 150 μL of PBS + 100 mM sucrose into the gaskets. To minimize evaporation, we place the gaskets and a water-saturated Kimwipe in a sealed 150 mm diameter Petri dish. The films were allowed to hydrate for 2 h on a laboratory bench at room temperature. For assembly at 37 °C, we preheated the buffers to 37 °C in a water bath. The films were allowed to hydrate for 2 h on a hotplate set to 37 °C. To prevent evaporation, we covered the gaskets with glass coverslips and placed the gaskets and a water-saturated Kimwipe in a sealed 150 mm diameter Petri dish.

### Fructose-Doped Lipid Method

Following a previously published protocol,^20^ we prepared 1 mM (0.785 mg mL^–1^) DOPC/TopFluor-Chol at 99.5:0.5 mol % in chloroform and 20 mM fructose dissolved in neat methanol. We mixed 50 μL of 1 mM 99.5:0.5 mol % DOPC/TopFluor-Chol in chloroform with 25 μL of 20 mM fructose in methanol to create a 2:1 chloroform/methanol working solution. We applied 15 μL of the solution onto the coverslip and placed the coverslip in a vacuum chamber for 1 h. The dried lipid film was then hydrated in 100 mM sucrose in PBS.

### Procedure for Harvesting the GUVs

Harvesting GUVs from the substrate was conducted as previously described.^41^ Briefly, the GUVs were harvested by pipetting 100 μL of the hydrating solution with a cut 1000 μL pipet tip on six different regions of the lipid-coated surface to cover the whole area. We aspirated all the GUV-containing liquid for the seventh time and transferred the liquid into a 0.5 mL Eppendorf tube. The final sample volume was ∼150 μL. Aliquots were taken immediately for imaging.

### Confocal Microscopy of Harvested Vesicles

Imaging of harvested vesicles was conducted as previously described.^41^ Briefly, we constructed imaging chambers by placing PDMS gaskets with a square opening (width × length × height = 6 × 6 × 1 mm^3^) on glass microscope slides. Before use, we passivated the chamber with a solution of 1 mg mL^–1^ casein in PBS to prevent the rupture of GUVs on the surface of a bare glass. Chambers were thoroughly rinsed with ultrapure water after passivation. We filled the passivated chamber with 58 μL of a 100 mM solution of glucose in PBS and evenly distributed a 2 μL aliquot of harvested GUV suspension into the 100 mM glucose in PBS solution by repeatedly pipetting 2 μL of the mixed suspension in glucose. We allowed the GUVs to sediment for 3 h in a sealed 150 mm Petri dish with a water-saturated Kimwipe to prevent evaporation before imaging. We captured images using an upright confocal laser-scanning microscope (LSM 880, Axio Imager.Z2m, Zeiss, Germany), using a 488 nm argon laser and a 10× Plan-Apochromat objective with a numerical aperture of 0.45. We imaged using an automated tile scan routine (64 images [850.19 μm × 850.19 μm (3212 pixels × 3212 pixels)]) to capture the entire area of the chamber. The routine used an autofocus feature at each tile location. Out-of-focus tiles were imaged manually. The pinhole was set at 15.16 Airy units, which gave a confocal slice thickness of 79.3 μm.

### Imaging the Surface of Hydrated Lipid Films

We captured images of the surfaces using an upright confocal laser-scanning microscope (LSM 700, Axio Imager.Z2m, Zeiss, Germany), a diode-488 nm laser, and a 10× Plan-Apochromat objective with a numerical aperture of 0.45. The frame size was 2048 pixels × 2048 pixels. The pinhole was set to 1 Airy Unit, which gave a confocal slice thickness of 5.9 μm. Images were selected to be representative of the whole surface.

### Image Processing and Analysis

We conducted image processing and analysis as previously described.^41^ Briefly, we used a custom MATLAB (Mathworks Inc., Natick, MA) routine to analyze the confocal tile scan images. The routine segmented fluorescent objects from the background. To obtain the diameters and mean intensities of the objects, we used the native regionprops function. We used the coefficient of variance of the intensities to select GUVs from the detected fluorescent objects. All images were inspected after automated segmentation, and erroneously segmented objects were manually corrected.

### Statistical Analysis

All statistical analyses were performed using MATLAB. We conducted one-way balanced analysis of variance (ANOVA) in MATLAB to determine the statistical significance of the mean yields obtained for the different compounds. We conduct a posthoc Tukey’s honestly significant difference (HSD) to determine the statistical significance of the differences in the mean between pairs of surfaces. To compare the statistical significance of the difference of temperature on the yields, we conduct Student’s *t*-tests.

## Supporting Information Available

Supporting text describing mathematical details of the molar yield calculations and free energy model; figures showing: histograms of GUV diameters from different substrates; confocal images of harvested, free-floating GUVs, and buds on the surface of the substrates; tables showing: compounds tested, *p*-values for statistical tests, concentrations of salts in buffers, and calculated Debye screening lengths (PDF)
