# Supplementary material for: Osmotic Pressure Enables High-Yield Assembly of Giant Vesicles in Solutions of Physiological Ionic Strengths
Source: Langmuir. 2023 Apr 6;39(15):5579–90. doi: 10.1021/acs.langmuir.3c00457 (PMC10116648; doi:10.1021/acs.langmuir.3c00457)
Supplement: Supplementary file 1 — la3c00457_si_001.pdf [file la3c00457_si_001.pdf]

Supporting Information

Osmotic pressure enables high yield assembly of giant vesicles in solutions of physiological ionic strengths

Alexis Cooper[a], Vaishnavi Girish[b], and Anand Bala Subramaniam [b*]

[a] Department of Chemistry and Biochemistry, University of California, Merced, Merced, CA 95343, United States.

[b] Department of Bioengineering, University of California, Merced, Merced, CA 95343, United States.

*Email: asubramaniam@ucmerced.edu

Supporting Text

Determining the effect of dissolved polymers on the sedimentation of GUVs. High concentrations of polymeric molecules in solution could in principle decrease the sedimentation velocity of the GUVs by increasing the viscosity of the solution. Polymers are present at low concentrations in the imaging chambers.

To demonstrate, we calculate the maximum possible concentration of polymeric molecules in the solution containing the harvested GUVs (assuming all the polymer molecules dissolve) using Equation S1. We assume all solutions have the density of water $\rho = 1\ \mathrm{g/mL}$.

$$w_{dissolved}^{max} = \frac{w_{ps} m_{ps}}{A_{coverslip}} \frac{A_{chamber}}{m_h} \tag{S1}$$

In this equation, $w_{dissolved}^{max}$ is the maximum concentration (w/w %) of dissolved polymer in the hydrating solution, w_{ps} is the concentration of the polymer solution (we used 1 w/w %) deposited on the glass coverslip, m_{ps} is the mass of the polymer solution (we used 0.3 mL ≈ 0.3 g) deposited on the glass coverslip, $A_{coverslip}$ is the area of the coverslip (484 mm^2), $A_{chamber}$ is the area of the hydration chamber (113 mm^2), and m_h is the mass of the hydrating buffer (we used 0.15 mL ≈ 0.15 g). $w_{dissolved}^{max} \approx 0.47$ w/w %. Further, in the imaging chamber, the harvested vesicle suspension is diluted 30 times in the sedimentation buffer. The sedimentation buffer is devoid of polymer molecules. Thus, the maximum concentration of dissolved polymeric molecules in the imaging chamber in the extreme case of complete dissolution is 0.016 w/w%. Note that in actuality, the polymers that we use are only partially soluble, thus the concentration of dissolved polymeric molecules is expected to be much lower than this maximum value.

The imaging chamber that we used has a height of 1000 µm. The vesicles are initially present in all locations in the chamber since they are well-mixed. To test for differences, after 3 hours, we image the solution at the imaging plane (5 µm above the coverslip) and the bulk at 100

μm and 900 μm above the imaging plane for GUVs composed of DOPC harvested from bare glass without any assisting compounds and when LGT agarose is used as the assisting compound. In both cases, we observe a large number of GUVs in the imaging plane and no GUVs in the locations in the bulk (Fig. S1). Our results confirm that the low concentration of polymeric molecules, ≪ 0.016 wt %, in the solution in the imaging chamber does not have any measurable effect on the sedimentation behavior of GUVs.

Calculation of molar yield from the literature. Reference[1] reports that the total number of GUVs with diameters between 10 μm and 80 μm in a 20 μL aliquot from a harvested volume of 500 μL is 63 ± 14. The molar yield is defined as the mols of lipids in GUV membranes divided by the mols of lipids used to assemble the GUVs[2]. The mols of lipid initially used in Reference 1 was 1×10^{-9} mols [1]. We estimate the maximum possible mols of lipids in the harvested vesicles reported in Reference 1 using Equation S2.

$$moles\ of\ lipid = \frac{2\pi d^2 N_{GUV} V_h}{A_{hg} N_A V_{al}} \qquad \text{(S2)}$$

Here N_{GUV} is the number of GUVs, taken as 77, d_{GUV} is the diameter of the GUVs, taken to be 80 μm, A_{hg} is the headgroup area of the lipid which for DOPC is 72.4×10^{-4} μm^2, $N_A = 6.023 \times 10^{23}$ is Avogadro's number, V_h is the harvested volume which is 500 μL, and V_{al} is the aliquot volume that was quantified which is 20 μL. Choosing the largest diameter of GUV reported and the upper range of GUVs counted in the experiment ensures that we are calculating the maximum possible mols of lipids harvested as GUVs from Reference 1.

Dividing the mols of lipid in the harvested vesicles with the mols of lipids initially used gives a maximum molar yield of 6.0×10^{-4} % for Reference 1. Notably, the diameters of GUVs quantified in Reference 1 were limited to 10 μm ≤ d < 100 μm [1]. When we similarly limit our range, we find that the yield of GUVs from the fructose-doped technique that we performed is 3.9

$\times$ 10^{-2} %. This result is ~ 2 orders of magnitude greater than that calculated from Reference 1. Nevertheless, both these values show that the fructose-doped method produces a very low yield of GUVs in physiological salt solutions.

Energy for forming spherical buds from flat surfaces. The free energy of a budding membrane that is templated on a surface is modeled by Equation S3 [2].

$$\mathrm{E} = \int_{\boldsymbol{S}} d\boldsymbol{S}\left\{\frac{\kappa_b}{2}(2H - H_0)^2 + \kappa_G G + \kappa_a\right\} + \int_V dV\,\Delta\mathrm{P} + \int_{\boldsymbol{S}} d\boldsymbol{S}\,\{\xi\,(\boldsymbol{h})\} \qquad \text{(S3)}$$

In this equation, κ_b is the bending modulus, κ_G is the Gaussian bending modulus, $H = \frac{1}{2}(\kappa_1 + \kappa_2)$ is the mean curvature where κ_1 and κ_2 are the principal curvatures on the surface, $G = \kappa_1\kappa_2$ is the Gaussian curvature, H_0 is the spontaneous curvature, κ_a is the area expansion modulus, $\Delta\mathrm{P}$ is the difference in osmotic pressure, and $\xi(\boldsymbol{h})$ is the microscopic interaction potential normal to the surface of the membrane. The magnitude of $\xi(\boldsymbol{h})$ depends on the distance, $\boldsymbol{h}$, between the membrane and the surface[3]. These quantities are integrated as appropriate over the surface, $\boldsymbol{S}$, of the membrane, or the volume, V, that the membrane encloses.

Equation S3 can be simplified by noting that $H_0 = 0$ for a symmetric bilayer and that there is no change in the Gaussian curvature for spherical buds that remain attached to the surface[2]. We further simplify by replacing the microscopic interaction potential, $\xi(\boldsymbol{h})$, with an effective adhesion contact potential, ξ [2]. We obtain Equation S4 by i) integrating Equation S3 to obtain the energy for State 1, the geometry of a flat disk of radius R_d, the energy for State 2, the geometry of a spherical bud of radius $R_b = \frac{R_d}{2}$, and ii) subtracting the energy of State 1 from State 2.

$$\Delta\mathrm{E} = 8\pi\kappa_B + 2\pi R_d\lambda - \pi {R_d}^2\xi + \Delta P\Delta V \qquad \text{(S4)}$$

The second term on the RHS of Equation S4 introduces a constraint for a section of the membrane to transition into a spherical bud at a constant area. If there is a lipid source, the membrane can transition without requiring breaks by recruiting lipids from the source[3]. In the absence of a lipid source, the membrane must form breaks, with an edge energy λ, to allow the lipids to reconfigure to form a spherical bud. In the main manuscript, for simplicity, we assume that the membrane has a source and does not break during budding, thus $\lambda = 0$. For gentle hydration on bare glass, $\Delta P = 0$. For polymer-coated surfaces ΔP depends on the concentration of dissolved polymer in the interlamellar space of the bilayer stack on the surface.

Estimate of the osmotic pressure contribution of the dissolved polymer. The behavior of the polymer and lipid is expected to be far from ideal due to the small length scales, the high concentrations, the large molecular weight of the polymer, and the time dependent process of lipid hydration and polymer dissolution. The polymers appear to interact with the lipid, which further changes the behavior. To make progress, we consider a simplified scaling argument. We assume ideal behavior and use characteristic length and energy scales to determine if the osmotic pressure exerted by the dissolving polymer at the concentrations that we use in our experiments can overcome an increase in adhesion between the membranes. Specifically, we estimate the concentration of dissolved polymer that is needed in the interlamellar space of the bilayer stacks so that the contribution of the osmotic pressure results in no change in the free energy between polymer-free budding in low salt solutions and polymer-assisted budding in salty solutions.

We assume that the effects of the salt and polymers on the edge energy, λ, and the bending rigidity, κ_B, to be negligible. The change in energy in the low-salt condition, ΔE_{LS} and salty condition, ΔE_{HS} is given by Equations S5 and S6.

$$\Delta \mathrm{E}_{\mathrm{LS}} = 8\pi\kappa_B + 2\pi R_d\lambda - \pi {R_d}^2\xi_{LS} \tag{S5}$$

$$\Delta E_{HS} = 8\pi\kappa_B + 2\pi R_d\lambda - \pi {R_d}^2\xi_{HS} + \Delta P\Delta V \quad \text{(S6)}$$

In these equations, ξ_{LS} is the adhesion energy in the low salt solution, ξ_{HS} is the adhesion energy in the salty solution, ΔP is the osmotic pressure difference, assumed to be constant, and ΔV is the change in volume.

The change in volume from a disk-shaped bilayer on the surface with radius R_d and interlamellar spacing height, z to a spherical bud of radius R_b is given by Equation S7a

$$\Delta V = \frac{1}{6}\pi R_d^3 - \pi R_d^2 z \quad \text{(S7a)}$$

We used $R_b = \frac{R_d}{2}$ for the transition between a disk to a spherical bud at a constant surface area to express Equation S3a in terms of R_d.

For an ideal small molecule osmolyte at low concentrations, ΔP is related to the concentration of the osmolyte, c by Equation S7b.

$$\Delta P = cRT \quad \text{(S7b)}$$

In this equation, R is the ideal gas constant, and T is the temperature.

We obtain Equations S7c and S7d which satisfies the condition that there be no change in energy between assembly of GUVs in the low salt solution and the salty solution.

$$\Delta\Delta E = \Delta E_{HS} - \Delta E_{LS} = 0 \quad \text{(S7c)}$$

$$\Delta P = -\frac{\pi R_d^2}{\Delta V}(\xi_{HS} - \xi_{LS}) \quad \text{(S7d)}$$

We substitute Equation S7a and S7b into equation S7d to get Equation S8. Equation S8 relates the concentration of the polymer in the interlamellar space that is needed to balance the increase in the adhesion energy in the salty solution compared to the low salt solution.

$$c = -\frac{6(\xi_{HS} - \xi_{LS})}{RT(R_d - 6z)} \tag{S8}$$

We take $\xi_{LS} = 1 \times 10^{-6}\ \mathrm{J\ m^{-2}}$ for DOPC membranes in low-salt solutions and $\xi_{HS} = 1 \times 10^{-4}\ \mathrm{J\ m^{-2}}$ for DOPC membranes in salty solutions[3]. We use $z = 4\ \mathrm{nm}$ and $R_d = 1\ \mu\mathrm{m}$ for a GUV bud 1 μm in diameter. We get $c \approx 0.2407\ \frac{\mathrm{mol}}{\mathrm{m^3}} = 0.24$ mM. The use of the ideal expression for osmotic pressure in the dilute limit does not change our conclusion that low amounts dissolved polymer is sufficient to exert an osmotic pressure that balances the adhesion energy in high salt solutions. For macromolecular polymers at moderate concentrations, the osmotic pressure is often expressed as $\Delta P = (c + A_2(Mc)^2)RT$, where A_2 is the second virial coefficient and M is the molecular weight of the polymer[4]. The magnitude of the osmotic pressure, ΔP, is thus expected to be higher for polymers compared to small molecule solutes for the same dissolved concentration, c. A lower amount of dissolved polymer than predicted by Equation S8 will be sufficient to balance the increased adhesion between membranes in salty solutions.

We next consider the expected concentration of dry polymer, c_d, in the interlamellar volume of the bilayer stack. The mass of polymer molecules per unit area of substrate (0.003 g of the polymer spread over a coverslip area of $4.84 \times 10^{-4}\ \mathrm{m^2}$) is $6.2\ \frac{\mathrm{g}}{\mathrm{m^2}}$. We assume that upon hydration the polymer and lipid form uniform stacks composed of 5 lipid bilayers with an interlamellar spacing of 4 nm. The concentration of dry polymer in the stack with a molecular weight, $M = 120{,}000$ is $c_d = 2583\ \frac{\mathrm{mol}}{\mathrm{m^3}} = 2.58$ M. Thus, in this ideal calculation, approximately 0.009 % of the polymer must dissolve to form GUV buds in salty solutions.

Electrostatic interaction between membranes. The adhesion of membranes is affected by the presence of ions in solutions[5–7]. Ions promote adhesion by screening repulsive electrostatic interactions. The effectiveness of screening can be estimated using the Debye screening length[3]. Smaller screening lengths reflect a shorter range in which electrostatic repulsion is felt, thus allowing attractive van der Waals interaction to dominate[3]. We use Equation S9[3] to calculate the Debye screening length, $\frac{1}{\kappa_D}$.

$$\kappa_D = \sqrt{\frac{N_a e^2}{\varepsilon_0 \varepsilon k_B T} \sum_i [C]_i z_i^2} \tag{S9}$$

Here N_A is Avogadro's number, e is the elementary charge, $[C]$ is the concentration of ionic species i, z is the charge of ionic species i, ε_0 is the permittivity of free space, ε is the dielectric constant, k_B is the Boltzmann constant, and T is the absolute temperature.

We report the ionic composition of the buffers used for the experiments in Figure 9 in Table S10. We report the ionic species and the calculated Debye screening lengths in Table S11. Note that the solutions consisting of 100 mM sucrose and 140 mM KCl + 5 mM $CaCl_2$ are unbuffered. Our measurements showed that they have a pH of 5.5. This value of pH is consistent with the formation of carbonic acid when ultrapure water is in equilibrium with atmospheric carbon dioxide[8]. The screening length drops from 170 nm in the solution consisting of 100 mM sucrose to 0.75 nm in the solution consisting of PBS + 100 mM sucrose. The screening length is further reduced to 0.39 nm in the solution consisting of 600 mM NaCl + 100 mM sucrose. The calculated Debye screening lengths of PBS + 5 mM $MgCl_2$ and 140 mM KCl + 5 mM $CaCl_2$ are 0.73 nm and 0.77 nm respectively. Although the values of the Debye lengths are similar to those of PBS, adhesion is significantly enhanced because mM concentration of divalent cations can bind and neutralize surface charges and serve as ionic bridges between charges[9,10].

Supporting Figures

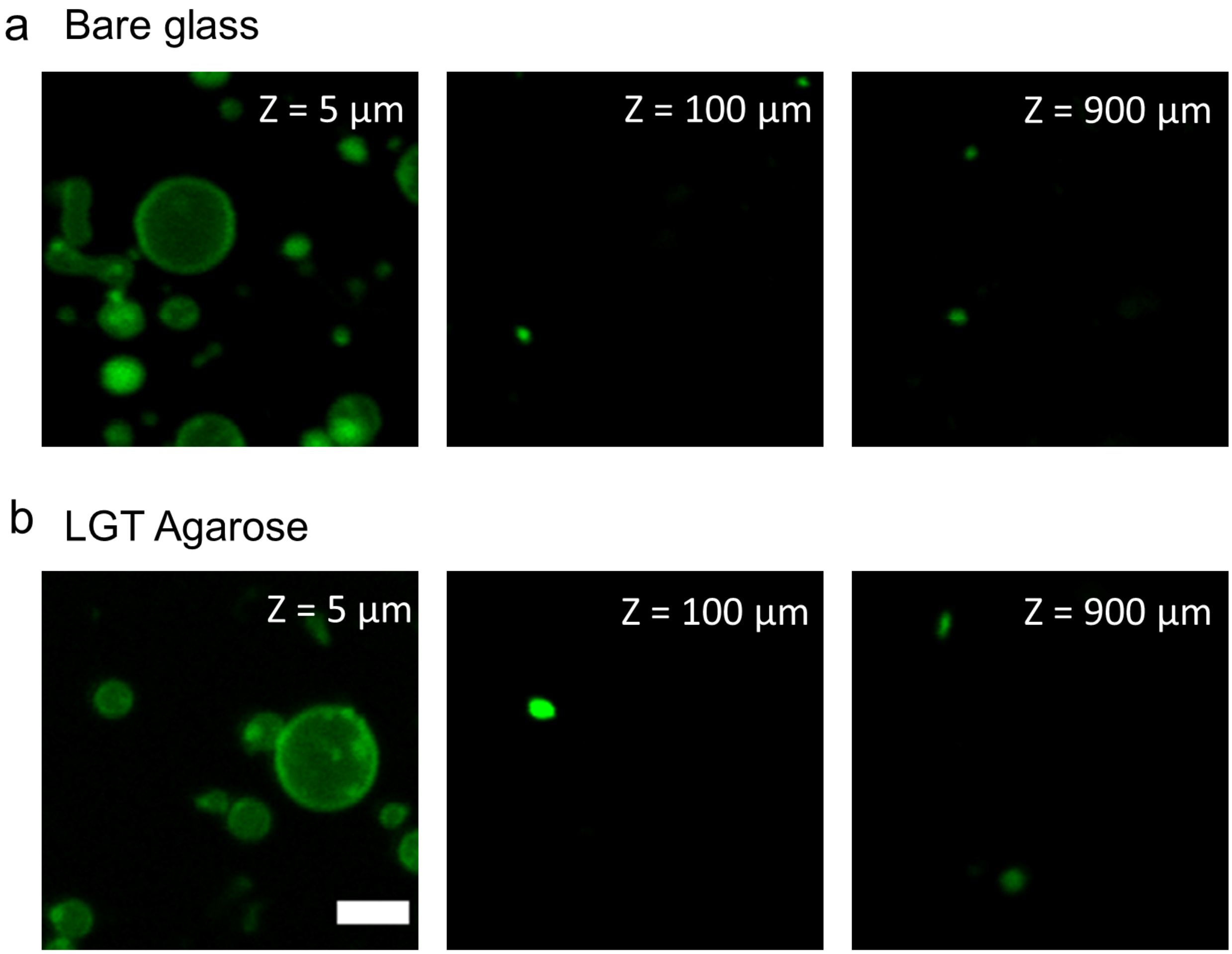

Figure S1. GUVs sediment in imaging chambers similarly in the presence and absence of polymers. Representative images at various *Z* planes of the imaging chamber after 3 hours of sedimentation. a) GUVs obtained from a bare glass surface without the use of assisting compounds. b) GUVs obtained when LGT agarose was used as the assisting compound. The Z position relative to the location of the surface of the bottom glass slide of the imaging chamber is indicated in the images. The scale bar is 10 µm.

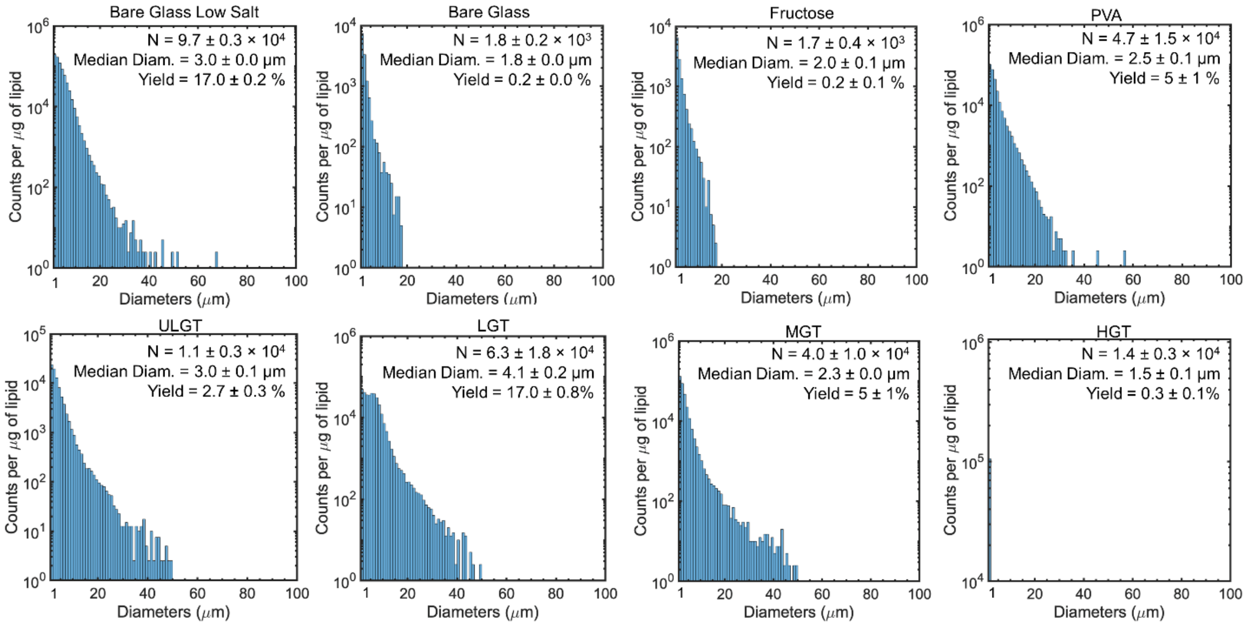

Figure S2. Histograms of GUV diameters of samples shown in Figure 2. Each histogram is the average of 3 independent repeats per sample. Note the logarithmic scale on the y-axis. Bin widths are 1 μm. The average number of GUVs counted, the median diameter, and the molar yield are shown in the text inset in the plots.

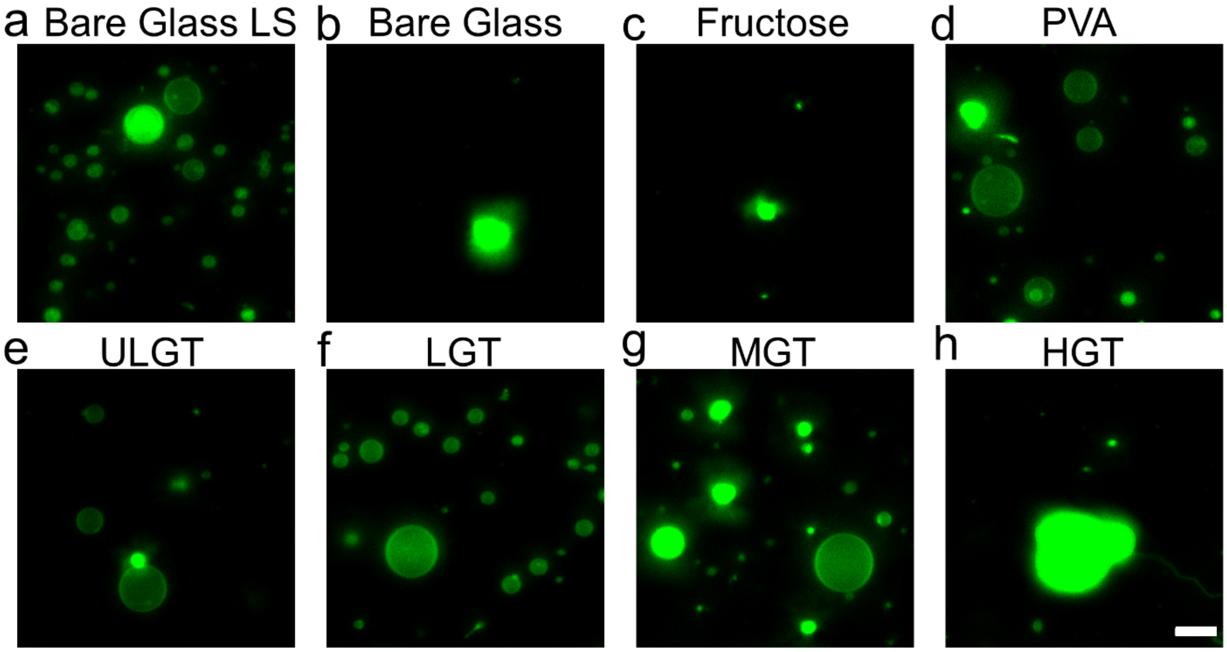

Figure S3. Representative images of the harvested objects for samples shown in Figure 2. Samples hydrated at 22 °C. Bright spots in all samples are lipid aggregates or MLVs. The scale bars are 15 μm.

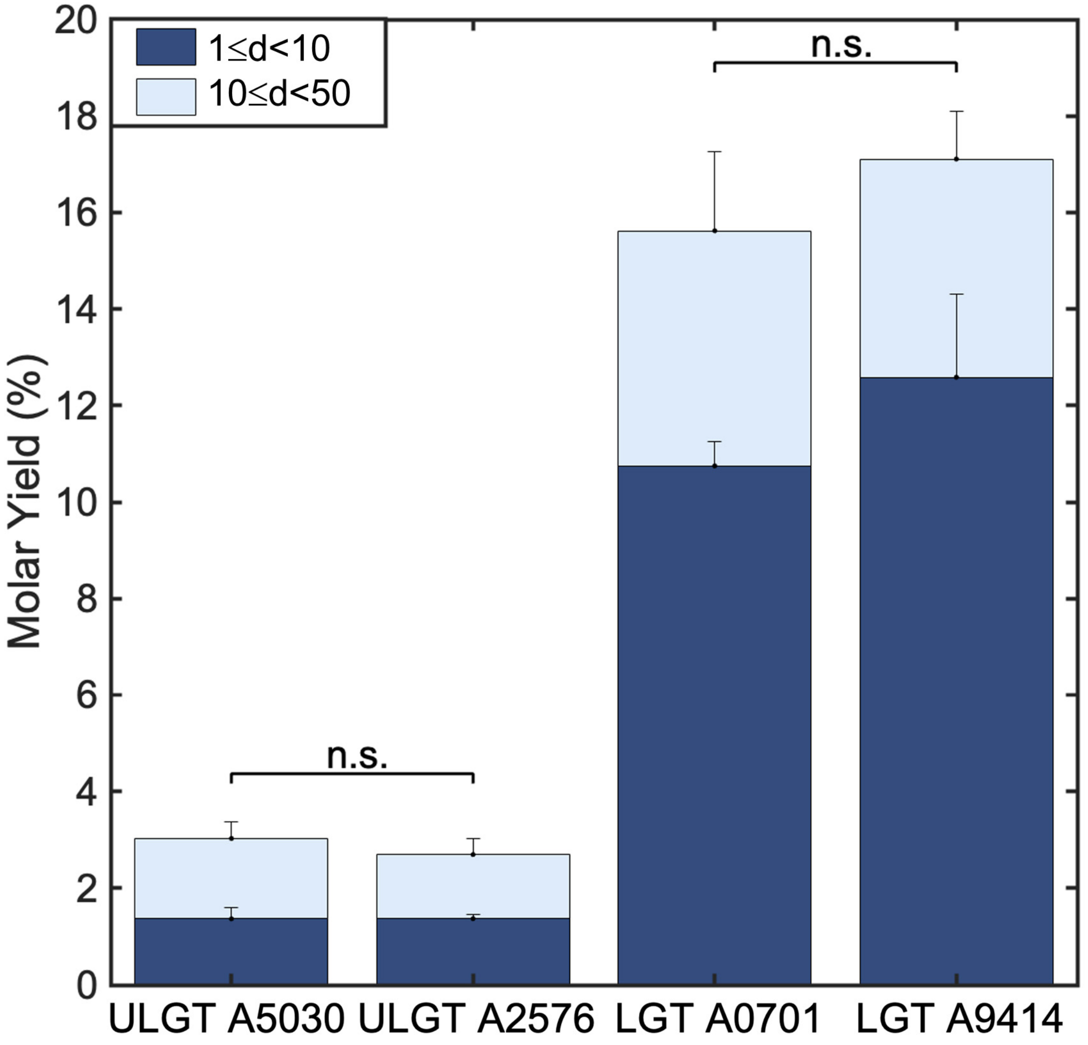

Figure S4. There is no significant difference in yields of GUVs obtained from ULGT and LGT agaroses with different catalog numbers (ultra-low gelling: A5030, A2576 and low gelling: A0701, A9414). Each bar is an average of 3 independent repeats per sample. Statistical significance determined by t-test. * = $p < 0.05$, ** = $p < 0.01$, *** = $p < 0.001$, ns = not significant. The data for A2576 and A9414 are from Figure 2.

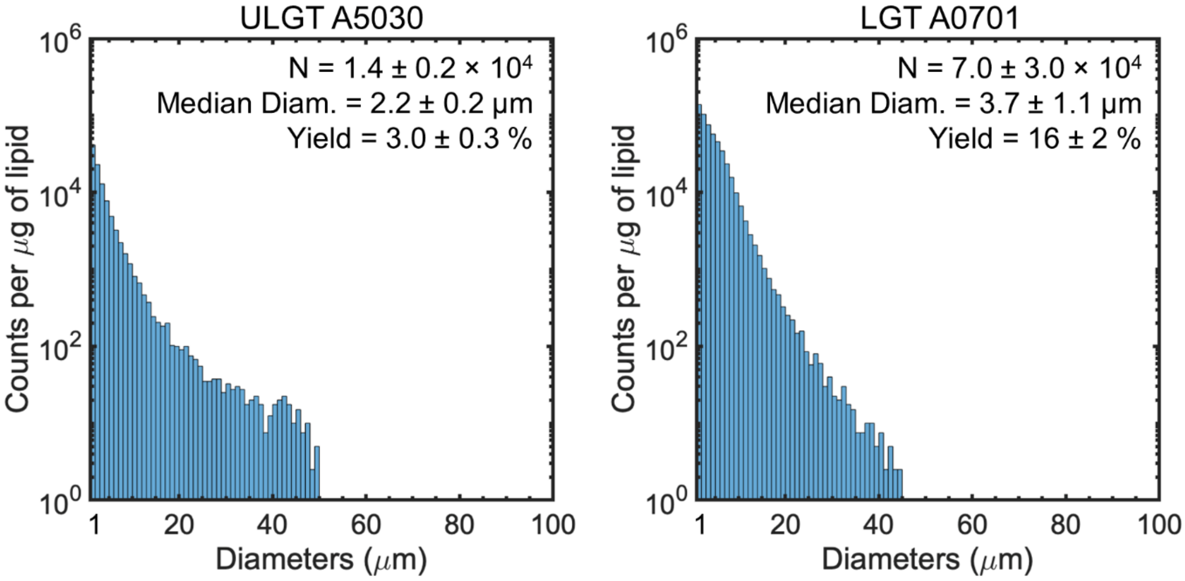

Figure S5. Histograms of GUV diameters of the samples shown in Figure S4. Each histogram is the average of 3 independent repeats per sample. Note the logarithmic scale on the y-axis. Bin widths are 1 µm. The average number of GUVs counted, the median diameter, and the molar yield are shown in the text inset in the plots.

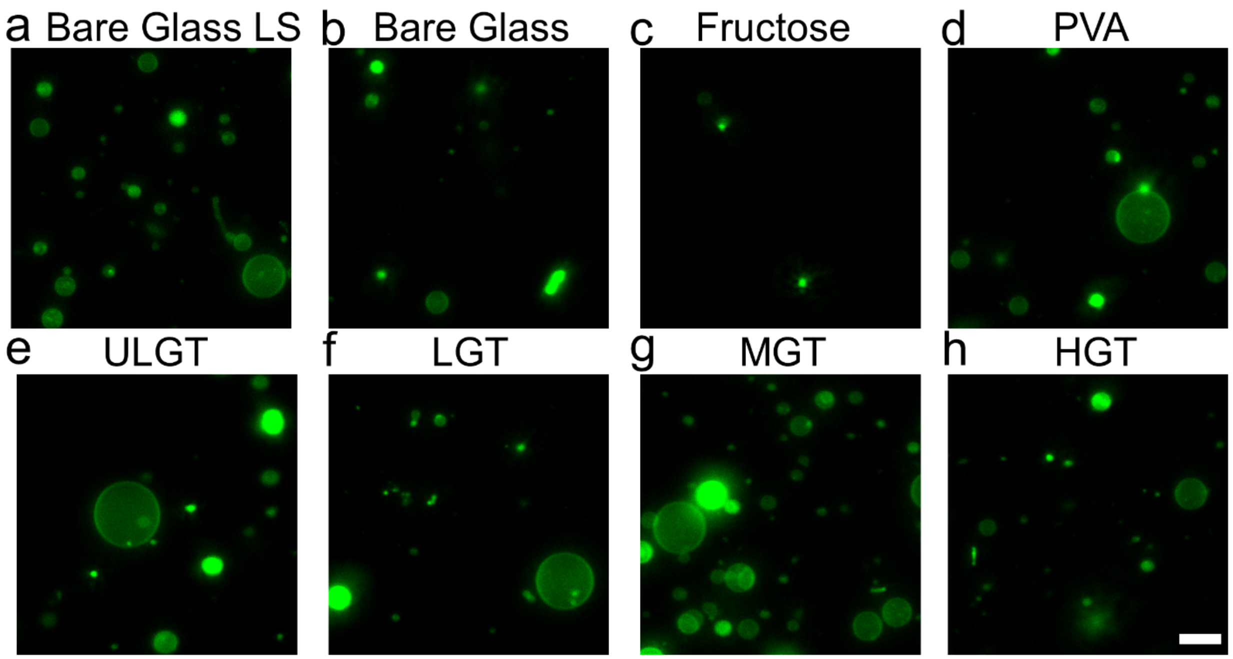

Figure S6. Representative images of the harvested objects for samples shown in Figure 3. Samples hydrated at 37 °C. Bright spots in all samples are lipid aggregates or MLVs. The scale bars are 15 µm.

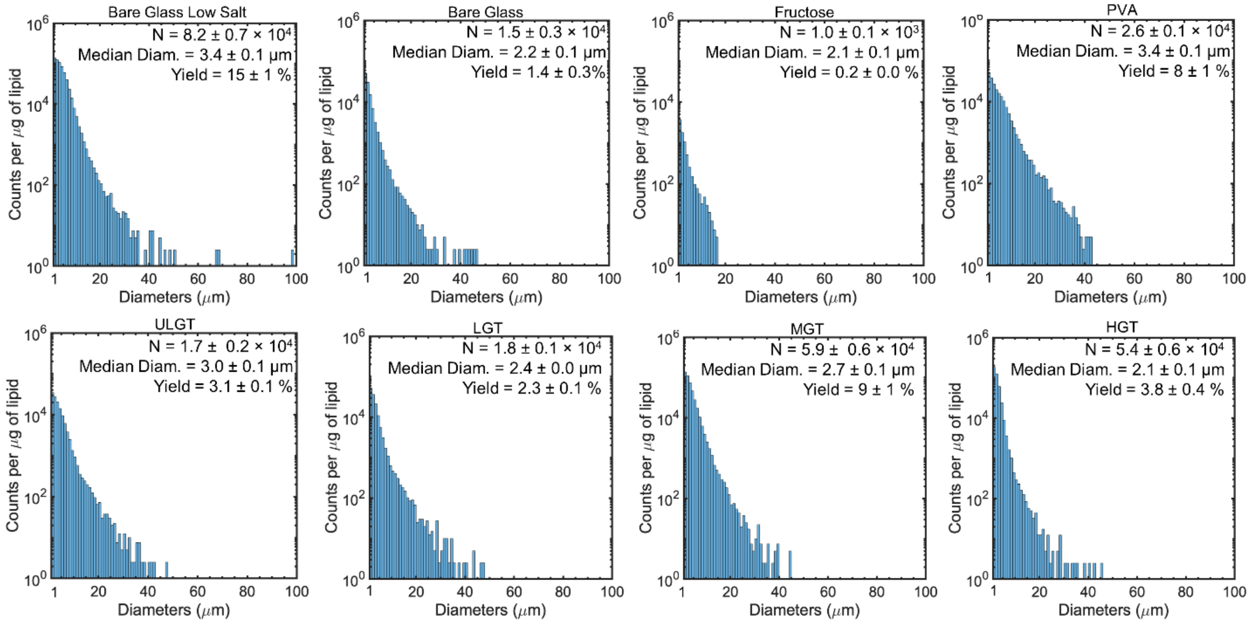

Figure S7. Histograms of GUV diameters of samples shown in Figure 3 for GUVs assembled at 37 °C. Each histogram is the average of 3 independent repeats per sample. Note the logarithmic scale on the y-axis. Bin widths are 1 µm. The average number of GUVs counted, the median diameter, and the molar yield are shown in the text inset in the plots.

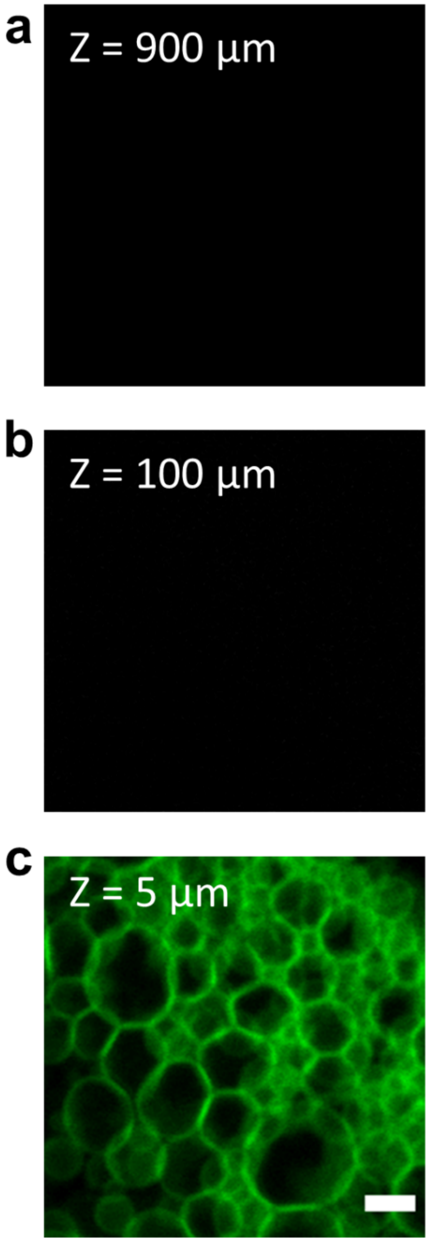

Figure S8. GUV buds remain attached to the surface in the absence of flow. Representative images after 2 hours of incubation at a) 900 μm and b) 100 μm above the LGT-agarose coated coverslip. Both regions had few to no floating structures. c) The surface of the agarose is covered with a high density of buds. The supporting glass coverslip is at $Z = 0$ μm. The scale bar is 10 μm.

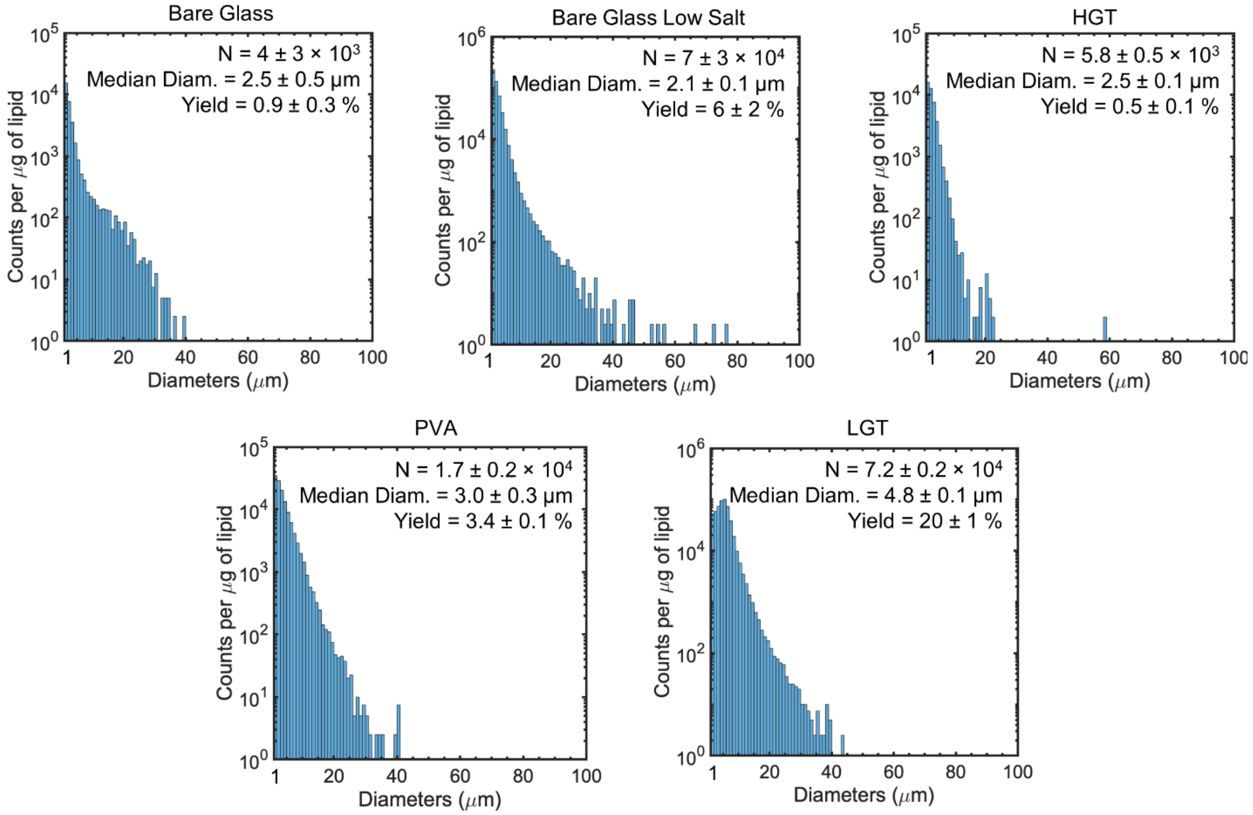

Figure S9. Histograms of GUV diameters of the samples shown in Figure S8a. The membranes of the GUVs are composed of a lipid mixture that minimally mimics the phospholipid composition of the exoplasmic leaflet of the mammalian cellular membrane (mammalian exoplasmic leaflet (MEL)). Each histogram is the average of 3 independent repeats per sample. Note the logarithmic scale on the y-axis. Bin widths are 1 µm. The average number of GUVs counted, the median diameter, and the molar yield are shown in the text inset in the plots.

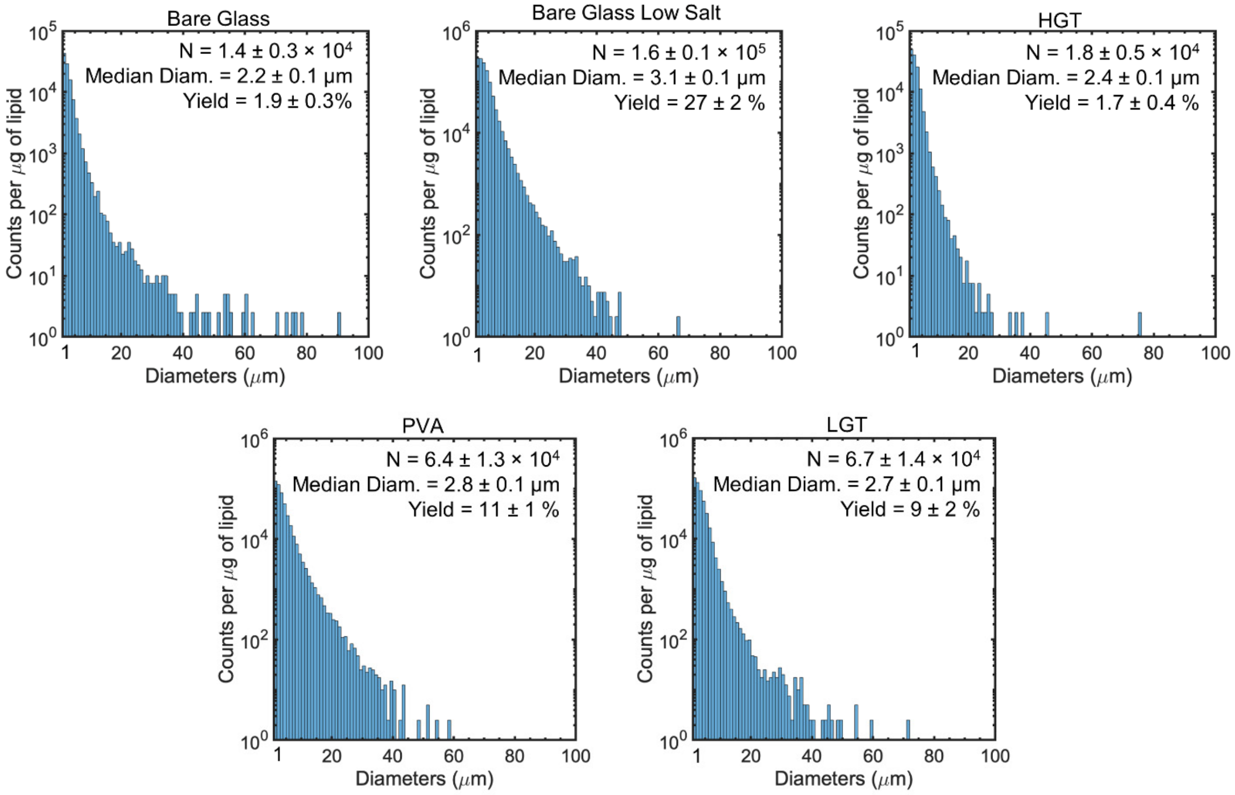

Figure S10. Histograms of GUV diameters of the samples shown in Figure S8b. The membranes of the GUVs are composed of a lipid mixture that minimally mimics the composition of the endoplasmic-reticulum-Golgi intermediate compartment (ERGIC) membrane. Each histogram is the average of 3 independent repeats per sample. Note the logarithmic scale on the y-axis. Bin widths are 1 μm. The average number of GUVs counted, the median diameter, and the molar yield are shown in the text inset in the plots.

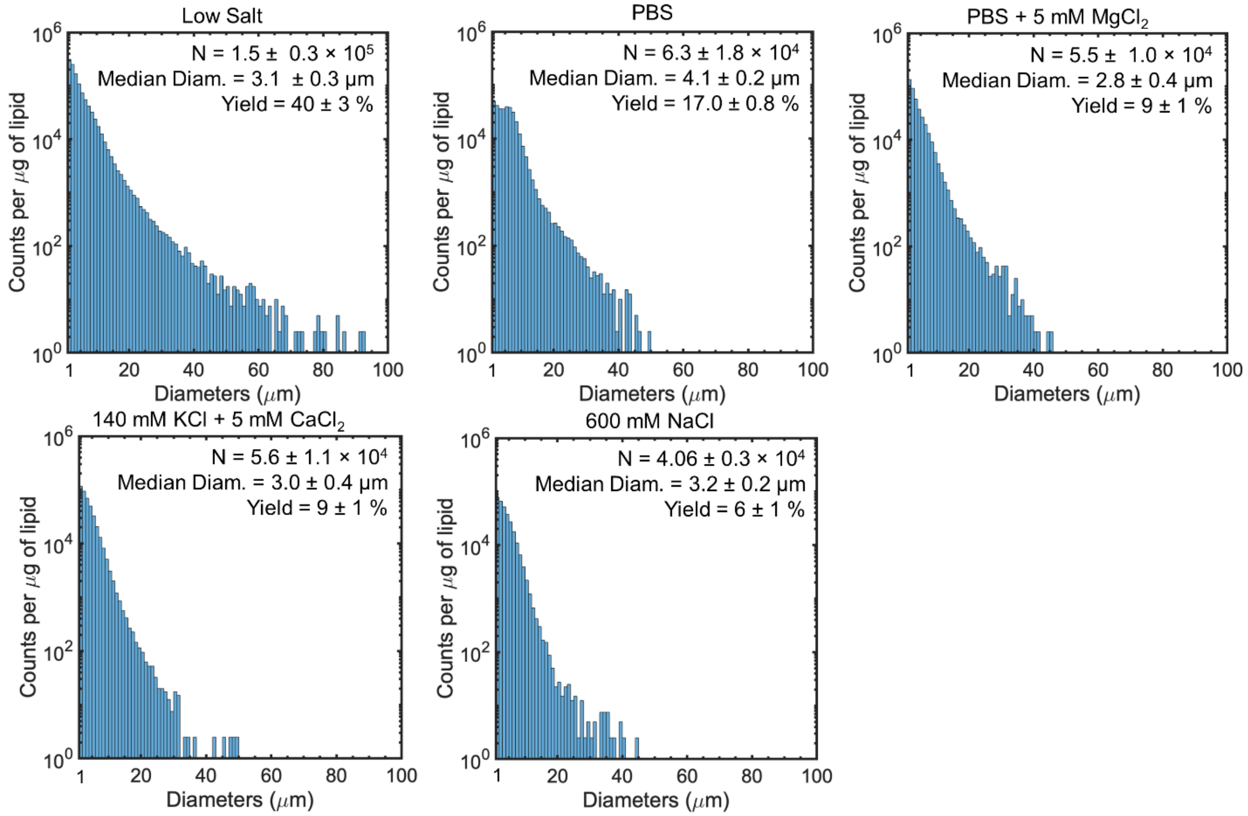

Figure S11. Histograms of GUV diameters of samples shown in Figure 9. The membranes of the GUVs are all composed of DOPC and the assisting compound is low gelling temperature (LGT) agarose. Each histogram is the average of 3 independent repeats per sample. Note the logarithmic scale on the y-axis. Bin widths are 1 µm. The average number of GUVs counted, the median diameter, and the molar yield are shown in the text inset in the plots.

Supporting Tables

Compound	Gel Point (°C)	Melting Point (°C)
**ULGT Agarose (Type IX-A, Catalog number: A2576)**	≤17 at 1.5%	≤60
**LGT Agarose (Catalog Number: A9414)**	~30	~65
**MGT Agarose (Type II-A, Catalog Number: A9918)**	36±1.5 at 1.5%	87±1.5
**HGT Agarose (Type VI-A, Catalog Number: A7174)**	41±1.5 at 1.5%	95±1.5
**ULGT Agarose (Type IX, Catalog Number: A5030)**	8-17 at 0.8%	≤60
**LGT Agarose (Type VII-A, Catalog Number: A0701)**	26±2.0 at 1.5%	≤65.5
**Polyvinyl alcohol**	85*	N/A
**Fructose**	N/A	N/A

Table S1. Melting and gelling temperatures of the compounds tested. *Glass transition temperature. Values for agarose are from [11], values for PVA is from [12].

Source	SS	*df*	MS	*F*	Probability>*F* (*p*-value)
Columns	651.351	6	108.558	149.654	7.04E-12
Error	10.156	14	0.725		
Total	661.506	20			

**Group 1**	**Group 2**	***p*-value**	**Significance**	**Comments**
Bare Glass LS	Bare Glass	6.732E-08	***	The addition of PBS resulted in a significant decrease of yield in comparison to gentle hydration in low salt.
Bare Glass LS	LGT	0.347	NS	The use of LGT agarose allows for assembly of GUVs in PBS with a molar yield not significantly different from bare glass in low salt.
Bare Glass	Fructose-doped	> 0.999	NS	Use of compounds in Group 2 was ineffective at increasing the yields of GUVs compared to gentle hydration in PBS.
Bare Glass	HGT	> 0.999	NS	
Bare Glass	ULGT	0.0326	*	Use of compounds in Group 2 was effective at increasing the yields of GUVs compared to gentle hydration in PBS.
Bare Glass	PVA	1.52E-04	***	
Bare Glass	MGT	1.59E-04	***	
Bare Glass	LGT	3.76E-08	***	
Fructose-doped	HGT	> 0.999	NS	Use of compounds in Group 2 was not effective at increasing the yields of GUVs compared to gentle hydration using fructose-doped lipid in PBS.
Fructose-doped	ULGT	0.0388	*	Use of compounds in Group 2 was effective at increasing the yields of GUVs compared to gentle hydration using fructose-doped lipid in PBS.
Fructose-doped	PVA	1.77E-04	***	
Fructose-doped	MGT	1.85E-04	***	
Fructose-doped	LGT	3.76E-08	**	

HGT	ULGT	0.0445	*	The difference in yield between HGT and compounds in group 2 is significant.
HGT	PVA	2.00E-04	***	
HGT	MGT	2.10E-04	***	
HGT	LGT	3.76E-08	***	
ULGT	PVA	0.0895	NS	The difference in yield between ULGT and compounds in group 2 not significant.
ULGT	MGT	0.0940	NS	
ULGT	LGT	3.77E-08	***	The difference in yield between ULGT and LGT is not significant.
PVA	MGT	0.999	NS	The difference in yield between PVA and MGT is not significant.
PVA	LGT	3.86E-08	***	The difference in yield between PVA and LGT is significant.
MGT	LGT	3.85E-08	***	The difference in yield between MGT and LGT is significant.

Table S2: ANOVA table and table of p-values from post hoc Tukey's HSD tests of the molar yields of GUVs assembled at 22 °C obtained from gentle hydration on bare glass in PBS (Bare Glass), fructose-doped lipid in PBS, and hydration on polymer films of PVA, ULGT agarose, LGT agarose, MGT agarose, and HGT agarose in PBS. Comparison of gentle hydration on bare glass in low salt (Bare Glass LS) and gentle hydration on bare glass in PBS (Bare Glass) was conducted separately from the ANOVA via Student's t-test. All solutions contain 100 mM of sucrose. * = $p < 0.05$, ** = $p < 0.01$, *** = $p < 0.001$, NS = not significant.

Source	SS	*df*	MS	*F*	Probability>*F* (*p*-value)
Columns	176.662	6	29.444	111.642	5.23E-11
Error	3.692	14	0.264		
Total	180.354	20			

**Group 1**	**Group 2**	***p*-value**	**Significance**	**Comments**
Bare Glass LS	Bare Glass	1.34E-04	***	The addition of PBS resulted in a significant decrease of yield in comparison to gentle hydration in low salt.
Bare Glass LS	LGT	1.58E-04	***	The use of LGT agarose at 37 °C results in a yield significantly lower than bare glass in low salt.
Bare Glass	Fructose-doped	0.129	NS	Use of compounds in Group 2 was ineffective at increasing the yields of GUVs compared to gentle hydration in PBS.
Bare Glass	LGT	0.436	NS	
Bare Glass	HGT	8.84E-04	***	Use of compounds in Group 2 was effective at increasing the yields of GUVs compared to gentle hydration in PBS.
Bare Glass	ULGT	0.0160	*	
Bare Glass	PVA	4.87E-08	***	
Bare Glass	MGT	3.90E-08	***	
Fructose-doped	HGT	1.06E-05	***	Use of compounds in Group 2 was effective at increasing the yields of GUVs compared to gentle hydration using fructose-doped lipid in PBS.
Fructose-doped	ULGT	1.15E-04	***	
Fructose-doped	PVA	3.86E-08	***	
Fructose-doped	MGT	3.78E-08	***	
Fructose-doped	LGT	3.39E-03	**	
HGT	ULGT	0.672	NS	The difference in yield between HGT and ULGT is not significant.
HGT	PVA	5.28E-06	***	

HGT	MGT	3.11E-07	***	The difference in yield between HGT and compounds in group 2 is significant.
HGT	LGT	0.0338	*	
ULGT	PVA	7.36E-07	***	The difference in yield between ULGT and compounds in group 2 is significant.
ULGT	MGT	8.77E-08	***	
ULGT	LGT	0.449	NS	The difference in yield between ULGT and LGT is not significant.
PVA	MGT	0.249	NS	The difference in yield between PVA and MGT is not significant.
PVA	LGT	1.15E-07	***	The difference in yield between PVA and LGT is significant.
MGT	LGT	4.53E-08	***	The difference in yield between MGT and LGT is significant.

Table S3: ANOVA table and table of *p*-values from post hoc Tukey's HSD tests of the molar yields of GUVs grown at 37 °C obtained from gentle hydration on bare glass in PBS (Bare Glass), fructose-doped lipid in PBS, and hydration on polymer films of PVA, ULGT agarose, LGT agarose, MGT agarose, and HGT agarose in PBS. Comparison of gentle hydration on bare glass in low salt (Bare Glass LS) and gentle hydration on bare glass in PBS (Bare Glass) was conducted separately from the ANOVA via Student's t-test. All solutions contain 100 mM of sucrose. * = $p < 0.05$, ** = $p < 0.01$, *** = $p < 0.001$, NS = not significant.

<table>
<tr><th>Group</th><th>p-value</th><th>Significance</th><th>Comments</th></tr>
<tr><td>LGT</td><td>1.40E-05</td><td>***</td><td>The increase in temperature from 22 °C to 37 °C results in a significant decrease in yields of GUVs.</td></tr>
<tr><td>HGT</td><td>2.20E-04</td><td>***</td><td rowspan="4">The increase in temperature from 22 °C to 37 °C results in a significant increase the in yields of GUVs.</td></tr>
<tr><td>Bare Glass</td><td>0.00480</td><td>**</td></tr>
<tr><td>MGT</td><td>0.0224</td><td>**</td></tr>
<tr><td>PVA</td><td>0.0314</td><td>*</td></tr>
<tr><td>ULGT</td><td>0.126</td><td>NS</td><td rowspan="3">The increase in temperature from 22 °C to 37 °C has no significant effect on the yields of GUVs.</td></tr>
<tr><td>Bare Glass LS</td><td>0.299</td><td>NS</td></tr>
<tr><td>Fructose-doped</td><td>0.632</td><td>NS</td></tr>
</table>

Table S4: Table of *p*-values from Student's t-tests of the molar yields of GUVs at 22 °C and 37 °C obtained from gentle hydration on bare glass in 100 mM sucrose (Bare Glass LS), gentle hydration on bare glass in PBS (Bare Glass), fructose-doped lipid in PBS, and hydration on polymers of PVA, ULGT agarose, LGT agarose, MGT agarose, and HGT agarose in PBS. All solutions contain 100 mM of sucrose. *= $p < 0.05$, ** = $p < 0.01$, *** = $p < 0.001$, NS = not significant.

Source	SS	*df*	MS	*F*	Probability>*F* (*p*-value)
Columns	212.636	3	70.879	40.563	3.474E-05
Error	13.979	8	1.747		
Total	226.615	11			

**Group 1**	**Group 2**	***p*-value**	**Significance**	**Comments**
Bare Glass LS	Bare Glass	1.554E-05	**	The addition of PBS resulted in a significant decrease of yield in comparison to gentle hydration in low salt.
Bare Glass	PVA	9.83E-05	***	Use of compounds in Group 2 resulted in a significant increase in yields of GUVs compared to gentle hydration in PBS.
Bare Glass	LGT	0.00130	**	
Bare Glass	HGT	0.998	NS	Use of compound in Group 2 was ineffective at significantly increasing yields of GUVs compared to gentle hydration in PBS.
PVA	LGT	0.0945	NS	The difference of yield between PVA and LGT is not significant
PVA	HGT	8.45E-05	***	The difference of yield between PVA and HGT is significant
LGT	HGT	0.00110	**	The difference of yield between LGT and HGT is significant

Table S5: ANOVA table and table of *p*-values from post hoc Tukey's HSD tests of the molar yields of GUVs composed of the ERGIC mixture grown at 22 °C obtained from gentle hydration on bare glass in 100 mM sucrose (Bare Glass LS), gentle hydration on bare glass in PBS (Bare Glass) and hydration on the polymer films of PVA, LGT agarose, and HGT agarose in PBS. Comparison of gentle hydration on bare glass in low salt (Bare Glass LS) and gentle hydration on bare glass in PBS (Bare Glass) was conducted separately from the ANOVA via Student's t-test.All

solutions contain 100 mM of sucrose. * = $p < 0.05$, ** = $p < 0.01$, *** = $p < 0.001$, NS = not significant.

Source	SS	*df*	MS	*F*	Probability>*F* (*p*-value)
Columns	775.721	3	258.574	1.448E+03	2.812E-11
Error	1.429	8	0.179		
Total	777.150	11			

**Group 1**	**Group 2**	***p*-value**	**Significance**	**Comments**
Bare Glass LS	Bare Glass	0.00714	**	The addition of PBS resulted in a significant decrease of yield in comparison to gentle hydration in low salt.
Bare Glass	HGT	0.738	NS	Use of compound in Group 2 was ineffective at significantly increasing yields of GUVs compared to gentle hydration in PBS.
Bare Glass	PVA	4.282E-04	***	Use of compounds in Group 2 resulted in a significant increase in yields of GUVs compared to gentle hydration in PBS.
Bare Glass	LGT	1.464E-03	***	
HGT	PVA	1.662E-04	***	The difference of yield between HGT and PVA is significant.
HGT	LGT	8.819E-14	***	The difference of yield between HGT and LGT is significant.
PVA	LGT	4.041E-12	***	The difference of yield between PVA and LGT is significant.

Table S6: ANOVA table and table of *p*-values from post hoc Tukey's HSD tests of the molar yields of GUVs composed of the MEL mixture grown at 22 °C obtained from gentle hydration on bare glass in 100 mM sucrose (Bare Glass LS), gentle hydration on bare glass in PBS (Bare Glass) and hydration on polymer films of PVA, LGT agarose, and HGT agarose in PBS. Comparison of gentle hydration on bare glass in low salt (Bare Glass LS) and gentle hydration on bare glass in PBS (Bare Glass) was conducted separately from the ANOVA via Student's *t*-test. All solutions contain 100 mM of sucrose. * = $p < 0.05$, ** = $p < 0.01$, *** = $p < 0.001$, NS = not significant.

Source	SS	*df*	MS	*F*	Probability>*F* (*p*-value)
Columns	214.874	2	107.437	59.687	1.096E-04
Error	10.800	6	1.800		
Total	225.674	8			

**Group 1**	**Group 2**	***p*-value**	**Significance**	**Comments**
DOPC	ERGIC	5.46E-04	***	The difference of yield between DOPC and ERGIC is significant.
DOPC	MEL	0.0868	NS	The difference of yield between DOPC and MEL is not significant.
ERGIC	MEL	1.08E-04	***	The difference of yield between ERGIC and MEL is significant.

Table S7: ANOVA table and table of *p*-values from post hoc Tukey's HSD tests of the molar yields of GUVs composed of the MEL mixture, DOPC mixture and ERGIC mixture assembled at 22 °C on using LGT agarose as the assisting compound in PBS. All solutions contain 100 mM of sucrose. * = $p < 0.05$, ** = $p < 0.01$, *** = $p < 0.001$, NS = not significant.

Source	SS	*df*	MS	*F*	Probability>*F* (*p*-value)
Columns	111.428	2	55.714	37.895	3.95E-04
Error	8.821	6	1.470		
Total	120.249	8			

**Group 1**	**Group 2**	***p*-value**	**Significance**	**Comments**
DOPC	ERGIC	0.00133	**	The difference of yield between DOPC and ERGIC is significant.
DOPC	MEL	0.358	NS	The difference of yield between DOPC and MEL is not significant.
ERGIC	MEL	4.43E-04	***	The difference of yield between ERGIC and MEL is significant.

Table S8: ANOVA table and table of *p*-values from post hoc Tukey's HSD tests of the molar yields of GUVs composed of the MEL mixture, DOPC mixture, and ERGIC mixture assembled at 22 °C on using PVA as the assisting compound in PBS. All solutions contain 100 mM of sucrose. * = $p < 0.05$, ** = $p < 0.01$, *** = $p < 0.001$, NS = not significant.

Source	SS	*df*	MS	*F*	Probability>*F* (*p*-value)
Columns	2275.586	4	568.897	196.320	1.87E-09
Error	28.978	10	2.898		
Total	2304.564	14			

**Group 1**	**Group 2**	***p*-value**	**Significance**	**Comments**
Low Salt	140 mM KCl + 5 mM $CaCl_2$	2.15E-08	***	Assembly using the buffer in Group 2 results in a significant decrease in yields of GUVs compared to assembly using the buffer in Group 1.
Low Salt	PBS + 5 mM $MgCl_2$	2.13E-08	***	
Low Salt	600 mM NaCl	1.77E-08	***	
Low Salt	PBS	1.43E-07	***	
PBS	600 mM NaCl	8.14E-05	***	
PBS	PBS + 5 mM $MgCl_2$	0.00124	**	
PBS	140 mM KCl + 5 mM $CaCl_2$	0.00137	**	
PBS + 5 mM $MgCl_2$	140 mM KCl + 5 mM $CaCl_2$	1.000	NS	Assembly using the buffer in Group 1 or Group 2 results in no significant difference in the yields of GUVs.
PBS + 5 mM $MgCl_2$	600 mM NaCl	0.228	NS	The addition of 5 mM $MgCl_2$ has an equivalent effect on the yield of GUVs as using 600 mM NaCl.
140 mM KCl + 5 mM $CaCl_2$	600 mM NaCl	0.206	NS	The addition of 5 mM $CaCl_2$ has an equivalent effect on the yield of GUVs as using 600 mM NaCl.

Table S9: ANOVA table and table of *p*-values from post hoc Tukey's HSD tests of the molar yields of GUVs assembled obtained using LGT agarose as the assisting compound in buffers of

different ionic compositions. All solutions contain 100 mM of sucrose. * = $p < 0.05$, ** = $p < 0.01$, *** = $p < 0.001$, NS = not significant.

Salt	100 mM sucrose	PBS+100 mM sucrose	PBS + 5 mM $MgCl_2$ +100 mM sucrose	140 mM KCl + 5 mM $CaCl_2$ + 100 mM sucrose	600 mM NaCl + 100 mM sucrose
NaCl	-	137 mM	137 mM	-	600 mM
KCl	-	2.7 mM	2.7 mM	140 mM	-
Na_2HPO_4	-	8 mM	8 mM	-	-
KH_2PO_4	-	2 mM	2 mM	-	-
$MgCl_2$	-	-	5 mM	-	-
$CaCl_2$	-	-	-	5 mM	-

Table S10. Table of the concentration of different salts in the hydration solutions used to gather the data in Figure 9.

Ionic species	100 mM sucrose	PBS+100 mM sucrose	PBS + 5 mM $MgCl_2$+100 mM sucrose	140 mM KCl + 5 mM $CaCl_2$+100 mM sucrose	600 mM NaCl+100 mM sucrose
Na^+	-	153 mM	153 mM	-	600 mM
K^+	-	4.7 mM	4.7 mM	140 mM	-
H^+	0.0032 mM	4×10^{-5} mM	4×10^{-5} mM	0.0032 mM	0.0032 mM
HCO_3^-	0.0032 mM	4×10^{-5} mM	4×10^{-5} mM	0.0032 mM	0.0032 mM
Cl^-	-	139.7 mM	149.7 mM	150 mM	-
HPO_4^{2-}	-	8 mM	8 mM	-	600 mM
$H_2PO_4^-$	-	2 mM	2 mM	-	-
Mg^{2+}	-	-	5 mM	-	-
Ca^{2+}	-	-	-	5 mM	-
**Debye Length**	170 nm	0.75 nm	0.73 nm	0.77 nm	0.39 nm

Table S11. Table of the concentration of ions in the hydration solutions. We assume complete dissociation of all ions. The last row shows the calculated Debye screening length.

Supporting References

1. Tsumoto, K., Matsuo, H., Tomita, M. & Yoshimura, T. Efficient formation of giant liposomes through the gentle hydration of phosphatidylcholine films doped with sugar. *Colloids Surfaces B Biointerfaces* **68**, 98–105 (2009).
2. Pazzi, J. & Subramaniam, A. B. Nanoscale Curvature Promotes High Yield Spontaneous Formation of Cell-Mimetic Giant Vesicles on Nanocellulose Paper. *ACS Appl. Mater. Interfaces* **12**, 56549–56561 (2020).
3. Israelachvili, J. N. *Intermolecular and Surface Forces: Third Edition*. *Intermolecular and Surface Forces: Third Edition* (2011). doi:10.1016/C2011-0-05119-0
4. Wang, S. C., Wang, C. K., Chang, F. M. & Tsao, H. K. Second virial coefficients of poly(ethylene glycol) in aqueous solutions at freezing point. *Macromolecules* **35**, 9551–9555 (2002).
5. Sun, Y., Lee, C. & Huang, H. W. Adhesion and Merging of Lipid Bilayers : A Method for Measuring the Free Energy of Adhesion and Hemifusion. *Biophysj* **100**, 987–995 (2011).
6. Marra, J. & Israelachvili, J. Direct Measurements of Forces between Phosphatidylcholine and Phosphatidylethanolamine Bilayers in Aqueous Electrolyte Solutions. 4608–4618 (1985). doi:10.1021/bi00338a020
7. Leckband, D. E., Helm, C. A. & Israelachvili, J. Role of Calcium in the Adhesion and Fusion of Bilayers. *Biochemistry* **32**, 1127–1140 (1993).
8. Mahnke, J., Stearnes, J., Hayes, R. A., Fornasiero, D. & Ralston, J. The influence of dissolved gas on the interactions between surfaces of different hydrophobicity in aqueous media. Part I. Measurement of interaction forces. *Phys. Chem. Chem. Phys.* **1**, 2793–2798 (1999).

9. Kucerka, N. *et al.* Cation-zwitterionic lipid interactions are affected by the lateral area per lipid. *Langmuir* (2021). doi:10.1021/acs.langmuir.0c02876

10. Kurakin, S. A., Ermakova, E. V., Ivankov, A. I., Smerdova, S. G. & Kučerka, N. The Effect of Divalent Ions on the Structure of Bilayers in the Dimyristoylphosphatidylcholine Vesicles. *J. Surf. Investig.* **15**, 211–220 (2021).

11. Sigma Aldrich. Agarose Product Information.

12. Hassan, C. M. & Peppas, N. A. Structure and applications of poly(vinyl alcohol) hydrogels produced by conventional crosslinking or by freezing/thawing methods. *Adv. Polym. Sci.* **153**, 37–65 (2000).
